# Increased transcriptional elongation and RNA stability of GPCR ligand binding genes unveiled via RNA polymerase II degradation

**DOI:** 10.1093/nar/gkae478

**Published:** 2024-06-06

**Authors:** Lijun Bao, Junyi Zhu, Tingxin Shi, Yongpeng Jiang, Boyuan Li, Jie Huang, Xiong Ji

**Affiliations:** Key Laboratory of Cell Proliferation and Differentiation of the Ministry of Education, School of Life Sciences, Peking-Tsinghua Center for Life Sciences, Peking University, Beijing 100871, China; Key Laboratory of Cell Proliferation and Differentiation of the Ministry of Education, School of Life Sciences, Peking-Tsinghua Center for Life Sciences, Peking University, Beijing 100871, China; Key Laboratory of Cell Proliferation and Differentiation of the Ministry of Education, School of Life Sciences, Peking-Tsinghua Center for Life Sciences, Peking University, Beijing 100871, China; Key Laboratory of Cell Proliferation and Differentiation of the Ministry of Education, School of Life Sciences, Peking-Tsinghua Center for Life Sciences, Peking University, Beijing 100871, China; Key Laboratory of Cell Proliferation and Differentiation of the Ministry of Education, School of Life Sciences, Peking-Tsinghua Center for Life Sciences, Peking University, Beijing 100871, China; Key Laboratory of Cell Proliferation and Differentiation of the Ministry of Education, School of Life Sciences, Peking-Tsinghua Center for Life Sciences, Peking University, Beijing 100871, China; Beijing Advanced Center of RNA Biology (BEACON), Peking University, Beijing 100871, China; Key Laboratory of Cell Proliferation and Differentiation of the Ministry of Education, School of Life Sciences, Peking-Tsinghua Center for Life Sciences, Peking University, Beijing 100871, China

## Abstract

RNA polymerase II drives mRNA gene expression, yet our understanding of Pol II degradation is limited. Using auxin-inducible degron, we degraded Pol II’s RPB1 subunit, resulting in global repression. Surprisingly, certain genes exhibited increased RNA levels post-degradation. These genes are associated with GPCR ligand binding and are characterized by being less paused and comprising polycomb-bound short genes. RPB1 degradation globally increased KDM6B binding, which was insufficient to explain specific gene activation. In contrast, RPB2 degradation repressed nearly all genes, accompanied by decreased H3K9me3 and SUV39H1 occupancy. We observed a specific increase in serine 2 phosphorylated Pol II and RNA stability for RPB1 degradation-upregulated genes. Additionally, α-amanitin or UV treatment resulted in RPB1 degradation and global gene repression, unveiling subsets of upregulated genes. Our findings highlight the activated transcription elongation and increased RNA stability of signaling genes as potential mechanisms for mammalian cells to counter RPB1 degradation during stress.

## Graphical Abstract

**Graphical Abstract:**
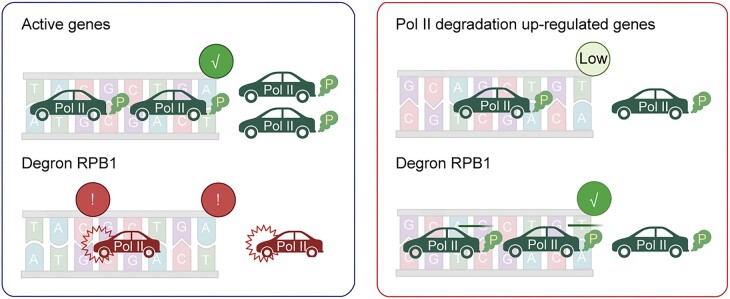

## Introduction

RNA Pol II consists of 12 subunits and is responsible for the expression of mRNA and lincRNA genes within cells (1–6). The subunits of RNA polymerases have been shown to play specific roles in Pol II CTD dephosphorylation, tissue-specific transcription, mRNA decay, and translation (7–9). Pol II transcription involves initiation through recruitment and CTD serine 5 phosphorylation, elongation with CTD serine 2 phosphorylation, and termination. Terminated Pol II is assumed to reinitiate transcription for another cycle (1–6). Throughout this process, it is assumed that Pol II transcription is coupled with various RNA processing activities such as 5′ RNA capping, splicing, RNA modification, cleavage, and polyadenylation (1–6). Furthermore, Pol II transcription is also recognized to coordinate with chromatin remodeling, chromatin structures, epigenetic modification, DNA replication, and DNA repair (10–16). However, the systematic identification of specific protein factors involved in DNA and RNA metabolic processes dependent on RNA polymerases at chromatin is still lacking in the field. This would serve as a starting point for further investigation into the mechanisms of crosstalk between RNA polymerases and DNA/RNA metabolic processes in the nucleus in the future.

Repression of RNA polymerase transcription has been found to suppress specific types of tumorigenesis and increase the lifespan in worms and flies (17–19). Interestingly, the transcription rate of Pol II also undergoes changes during aging (20–22). An inducible Pol II degradation system was previously established, following a comprehensive examination of its genome-wide effects, revealing that Pol II specifically regulates downstream tRNA transcription (23). Additionally, DNA damage has been shown to induce RPB1 degradation (24,25). Furthermore, under stress conditions such as chemical treatments (e.g. α-amanitin), UV exposure, serum starvation, and others, which typically lead to the degradation of the largest subunit RPB1 of Pol II and global transcription shutdown (26–28). Consequently, the repression of transcription is linked to many biological processes. However, detailed molecular investigations of Pol II degradation remain elusive, hindering the understanding of the impacts of Pol II degradation on various biological processes.

We utilized protein degradation system to target RNA polymerase and investigate the insights of gene expression and Pol II degradation under various stress conditions (29,30). Our findings revealed that the degradation of RPB1, the largest subunit of Pol II, resulted in the anticipated global transcription shutdown and decreased chromatin occupancy of RNA metabolic factors and chromatin regulators. Surprisingly, we observed that a subset of polycomb-bound short genes involved in GPCR ligand binding exhibited increased gene expression, reduced total Pol II occupancy, but increased occupancy of serine 2 phosphorylated Pol II (pSer2) and RNA stability for these genes. We conducted thorough analyses and experiments to further rule out alternative mechanisms, such as transcription rate, transcription termination, mRNA splicing, 3′ processing and RNA export. We then hypothesized that the decreased pool of RNA polymerase II in the nucleus triggers global transcriptional repression. Meanwhile, the presence of pSer2 at the short, polycomb-bound genes increased, thereby facilitating an increased number of elongating Pol II for these genes. Our proposed model suggests a dynamic interplay between global transcriptional repression and activation of transcriptional elongation and RNA stability within subsets of signaling genes, aimed at adapting to stress-induced RPB1 degradation.

## Materials and methods

### Cell culture

The V6.5 mouse embryonic stem cell line was provided by Richard Young's laboratory at the Whitehead Institute. Mouse embryonic stem cells (mESCs) were cultured in a medium consisting of 2i/mLIF. This medium was formulated with Dulbecco's modified Eagle's medium (DMEM)-high glucose (Simga#D5796-500mL), including 1 mM l-glutamine, 1× nucleosides (Sigma#ES-008), 1× nonessential amino acids (Millipore#TMS-001-C), 1× penicillin/streptomycin (Gibco#15140-122), 0.1 mM β-mercaptoethanol (Sigma#M3148), 3 μM CHIR99021 (Selleck#S1036) and 1 μM PD0325901 (Selleck#S1263). Additionally, the medium contained 1000 U/ml mouse leukemia inhibitory factor (mLIF) (Millipore#ESG1107) and was supplemented with 15% fetal bovine serum (FBS) (VivaCell#C04001-500). The cells were incubated at 37°C with 5% CO_2_ in a humidified incubator. To degrade the targeted proteins, the degron cells were treated with 1 μg/ml Dox for 12 h, followed by treatment with or without 500 μM IAA for an additional 3 or 12 h.

### Generation of RPB1-NTD degron Myc-RPB2 mESCs

The RPB1-NTD, RPB1-CTD and RPB2 degron mESCs were previously generated in our laboratory (29,30). For the construction of RPB1-NTD degron Myc-RPB2 cells, RPB1-NTD degron cells underwent transfection with RPB2 N-terminal targeting sgRNA, CRISPR/Cas9, and Myc-RPB2 donor plasmid. After two days, cells were subjected to selection with 250 μg/ml hygromycin B (Thermo Fisher Scientific#10687010) for one week, and colonies were collected for subsequent genotyping, sanger sequencing and Western blotting. The RPB1-NTD degron Myc-RPB2 mESCs were used for RPB1 degradation followed by RPB2 ChIP analyses due to the lack of high-quality antibodies. The RPB1-NTD degron in mESCs is used for RPB1 degradation in this study.

### Western blotting

The cells were washed with PBS and lysed directly in 2 × SDS-loading buffer (125 mM Tris–HCl, pH 6.8, 20% glycerol, 4% SDS, 0.2 M DTT, 0.2% Bromophenol blue). The lysates were then heated at 100°C for 15 min, the samples were then stored at –20°C. Following electrophoresis, proteins were transferred onto a PVDF membrane and subjected to immunoblotting using specific primary antibodies, which were diluted in 5% milk/TBST buffer (NaCl 8.8 g, 50 mM Tris–HCl (pH 8.0), 0.1% Tween) and incubated at 4°C overnight. The following primary antibodies were employed: anti-RPB1-NTD (Cell Signaling Technology#14958, 1:5000), anti-RPB2 (Santa Cruz#sc-166803, 1:200), anti-Phospho-Rpb1 CTD (pSer2) antibodies (Cell Signaling Technology#13499S, 1:5000), anti-Phospho-Rpb1 CTD (pSer5) antibodies (Cell Signaling Technology#13523S, 1:5000), anti-SNRNP70 antibodies (ABclonal#A6065 1:500), anti-Lamin B1 (Proteintech#12987-1-AP 1:2000) or anti-α-Tubulin (Proteintech#66031-1-Ig, 1:5000). On the next day, the membranes were washed with TBST buffer for three times on a shaker at room temperature, each time for 10 min, followed by incubation with secondary antibodies for 1 h at room temperature. Subsequently, the membranes underwent three washes and were captured using the AI 600 RGB imaging machine (GE).

### ChIP-seq and ChIP−qPCR

RPB1 and RPB2 degron cells were first collected, then 1 × 10^7^ cells mixed with 10% HEK293T cells were fixed with 1% formaldehyde, and they were incubated at room temperature for 10 min. For degron RPB1: RPB2 ChIP-seq, 10% HEK293T transfected with CTCF-Myc plasmids was used as spike-in. Following this, 0.125 M glycine was added to terminate formaldehyde crosslinking. The cells were washed twice with PBS, and the cell pellet was gently lysed on ice for 5 min using 0.5 ml pre-cooled NP-40 lysis buffer (10 mM Tris–HCl (pH 7.5), 150 mM NaCl, 0.05% NP-40). The cell lysate was added on top of the sucrose buffer cushion (containing 24% sucrose (wt/vol) in NP-40 lysis buffer) and centrifuged at 12 000 rpm for 10 min. The supernatant (representing the cytoplasmic fraction) was discarded, and the pellet was washed with PBS/1 mM EDTA. The nuclei pellets were resuspended in 0.5 ml glycerol buffer (20 mM Tris–HCl (pH 8.0), 75 mM NaCl, 0.5 mM EDTA, 0.85 mM DTT, 50% glycerol) and 0.5 ml nuclear lysis buffer (10 mM HEPES (pH 7.6), 1 mM DTT, 7.5 mM MgCl_2_, 0.2 mM EDTA, 0.3 M NaCl, 1 M urea, 1% NP-40). After incubating on ice for 2 min, the supernatant was discarded, and the pellet was washed twice with PBS/1 mM EDTA. The chromatin pellet was resuspended in 1 ml sonication buffer (20 mM Tris–HCl (pH 8.0), 150 mM NaCl, 2 mM EDTA (pH 8.0), 0.1% SDS, 1% Triton X-100) and 5 mM CaCl_2_, 1 × Cocktail protease inhibitor, 1 mM PMSF and 40 U MNase were added. The sample was incubated at 37°C, 700 rpm for 15 min. To inactivate MNase, 10 mM EDTA and 20 mM EGTA were added, and the mixture was placed on ice. Then, the pre-cooled Diagenode Bioruptor Plus sonicator was used with high power for 30 s on and 60 seconds off for 20 cycles. After sonication, the soluble chromatin fraction was taken, with 50 μl reserved as a positive control (input), and anti-RPB1 antibodies (Cell Signaling Technology#14958), anti-Phospho-Rpb1 CTD (pSer2) antibodies (Cell Signaling Technology#13499S), anti-Phospho-Rpb1 CTD (pSer5) antibodies (Cell Signaling Technology#13523S), anti-EZH2 antibodies (Cell Signaling Technology#5246S), anti-H3K4me3 antibodies (Millipore Sigma#04-745), anti-Myc Tag antibodies (Millipore Sigma#05-724) or anti-KDM6B/JMJD3 antibodies (Abcam#ab38113) were added to the soluble chromatin, and the sample was incubated overnight at 4°C. The next day, 40 μl Protein G magnetic beads (MedChemExpress #HY-K0204) were prewashed three times with sonication buffer and then incubated with the sample at 4°C for 3 h. The beads were washed sequentially with sonication buffer, high-salt wash buffer (20 mM Tris·HCl (pH 8.0), 500 mM NaCl, 2 mM EDTA, 0.1% SDS, 1% Triton X-100), and lithium chloride wash buffer (10 mM Tris·HCl (pH 8.0), 250 mM LiCl, 1 mM EDTA, 1% NP-40). The beads were washed once with TE buffer (1 mM EDTA, 10 mM Tris·HCl (pH 8.0)), and after the final wash, the remaining supernatant was removed. The beads were resuspended in 200 μl elution buffer (50 mM Tris·HCl (pH 8.0), 10 mM EDTA, 1% SDS) and incubated at 65°C, 1100 rpm for 30 min. 200 ng/ml Proteinase K (Thermo Fisher#25530049) was added, and the sample was incubated at 55°C for 1 h, followed by overnight incubation at 65°C to reverse crosslinking. The sample was mixed with an equal volume of phenol-chloroform, precipitated with ethanol, and suspended in 50 μl of pure water. Library construction was performed following the VAHTS Universal Pro DNA Library Prep Kit for Illumina (Vazyme#ND608) instructions, and sequencing was conducted using the Illumina platform, employing the PE150 sequencing strategy to generate 150-bp paired-end sequencing reads. For ChIP-qPCR, 1 μl of ChIP DNA was added directly to the qPCR reaction mixture which was performed using Taq Pro Universal SYBR qPCR Master Mix (Vazyme#Q712) following manufacturer's instructions (primers see Supplementary Table S7). Results were calculated using the ΔΔCt method.

### PolyA (+) RNA-seq and RT-qPCR

For RNA-seq, mESCs cells were treated with α-amanitin (10 μg/ml) for 24 h or UV-irradiated with 20 J/m^2^ and collected 12 h. About 1 × 10^6^ cells were collected with 10% *Drosophila* S2 cells and suspended in 0.5 ml TRIzol reagent. Total RNA was extracted according to the manufacturer's instructions. After sample qualification, 1–3 μg of total RNA from each sample served as the initial material for constructing a transcriptome sequencing library. Following the protocols of the VAHTS Universal V6 RNA-seq Library Prepkit for Illumina (Vazyme#NR604), various index tags were selected to establish the database. Following library qualification, sample pooling was performed based on the effective concentration of the library and the targeted data volume. Sequencing was conducted using the Illumina platform, employing the PE150 sequencing strategy to generate 150-bp paired-end sequencing reads.

For mRNA RT-qPCR analysis, about 1 × 10^6^ RPB1 and RPB2 degron cells were collected with 10% *Drosophila* S2 cells and suspended in 0.5 ml TRIzol reagent. Following total RNA extraction, an equal volume of total RNA was reverse transcribed into cDNA using oligo-dT primers according to instructions of HiScript III RT SuperMix for qPCR (Vazyme#R323). Then, Quantitative RT-PCR was performed using Taq Pro Universal SYBR qPCR Master Mix (Vazyme#Q712) following manufacturer's instructions (primers see Supplementary Table S7). Results were calculated using the ΔΔCt method.

### Chromatin-associated RNA-seq (ChAR-seq) and ChAR-RT-qPCR

Chromatin-associated RNA was isolated following a previously published protocol (31), with the buffer conditions described in the ChIP section. Briefly, approximately 1 × 10^7^ RPB1 degron cells and 10% *Drosophila* S2 cells were collected and lysed in NP-40 lysis buffer, followed by sucrose cushion precipitation. The cytoplasmic supernatants were discarded, and the nuclear pellets were resuspended in 0.5 ml glycerol buffer. To this, 0.5 ml nuclei lysis buffer was added, and the mixture was incubated on ice for 2 min. After centrifugation, the nucleoplasm supernatants were discarded, and the chromatin pellets were washed with PBS. RNA was then extracted from the chromatin pellets using TRIzol reagent, following the provided instructions. The RNA samples obtained were sent to Novogene for ribosomal RNA depletion and strand-specific library construction. Sequencing was conducted using the Illumina platform, employing the PE150 sequencing strategy to generate 150-bp paired-end sequencing reads. For ChAR-RT-qPCR, 2 μg of chromatin-associated RNA was reverse transcribed into cDNA using N9 random primers, following the instructions provided with HiScript III RT SuperMix for qPCR (Vazyme#R323). Then, qPCR was conducted and analyzed as described in RT-qPCR section (primers see Supplementary Table S7).

### Cytoplasmic and nuclear RNA-RT-qPCR

Approximately 1 × 10^7^ RPB1 NTD degron cells, which were lysed using NP-40 lysis buffer to release cytoplasmic and nuclear components. Following lysis, cytoplasmic supernatants and nuclear pellets were collected by centrifugation. To each fraction, 5% *Drosophila* S2 cells were added before proceeding to RNA extraction. Cytoplasmic RNA was then extracted from the supernatants using Trizol-LS reagent as per the manufacturer's instructions, while nuclear RNA was extracted directly from the pellets using Trizol reagent. Subsequently, 2 μg RNA from both fractions was reverse transcribed into cDNA using N9 random and oligo-dT primers, following the instructions provided with HiScript III RT SuperMix for qPCR (Vazyme#R323). Then, qPCR was conducted and analyzed as described in RT-qPCR section (primers see Supplementary Table S7).

### Transient transcriptome sequencing (TT-seq)

RPB1 and RPB2 degron cells were cultured in a 15 cm dish until reaching a cell density of 80–90%. Subsequently, 1 mM 4sU labeling was added, and the cells were incubated in the dark for 30 min. After removing the culture medium, 2 ml TRIzol was added to halt the labeling process. Following thorough lysis, cells were scraped from the culture dish, and the TRIzol-cell mixture was collected in a centrifuge tube, with the addition of 10% *Drosophila* S2 cells (pre-labeled with 100 μM 4sU for 12 h). Total RNA was extracted using the TRIzol method. The RNA was dissolved in two tubes with 400 μl of DEPC water each. Using the pre-cooled Diagenode Bioruptor Plus sonicator was used with high power for 30 s on and 30 s off for 10 cycles. After incubating at 65°C for 10 min and placing on ice for 5 min, RNA was diluted to 700 μl with DEPC water. Then, 100 μl of 10 × biotinylation buffer (100 mM Tris–HCl (pH 7.4), 10 mM EDTA) and 200 μl of 1 mg/ml Biotin-HPDP/DMF were added and mixed immediately. The reaction proceeded in the dark at room temperature for 2 h. An equal volume of chloroform was added to the biotinylation reaction, precipitated with isopropanol, and suspended in 100 μl of DEPC water. The RNA was then incubated at 65°C for 10 min and placed on ice for 5 min. For each sample, 40 μl of Dynabeads™ M-280 Streptavidin beads (Thermo Fisher Scientific#11205D) were pre-treated. The beads were washed with DEPC water containing 0.1 M NaOH and 50 mM NaCl, followed by two washes each with DEPC water containing 100 mM NaCl. Finally, the beads were washed three times each with binding buffer (10 mM Tris–HCl (pH = 7.4), 300 mM NaCl, 0.1% Triton X-100). The pre-treated beads were added to the biotinylated RNA and incubated at room temperature for 30 min. The beads were washed twice each with pre-cooled high-salt wash buffer (50 mM Tris–HCl (pH 7.4), 2 M NaCl, 0.5% Triton X-100), followed by two washes each with of binding buffer, and finally one wash with low-salt wash buffer (5 mM Tris–HCl (pH = 7.4), 0.1% Triton X-100). After extraction with TRIzol reagent, the purified RNA was used for library preparation with chain-specific library construction which was performed according to the VAHTS Universal V8 RNA-seq Library Prep Kit for Illumina (Vazyme#NR605) instructions, and sequencing was conducted using the Illumina platform, employing the PE150 sequencing strategy to generate 150-bp paired-end sequencing reads.

### 4sU labeling pulse-chase RNA-seq and 4sU labeling pulse-chase RNA-RT-qPCR

RPB1 degron cells were cultured in a 15 cm dish until reaching a cell density of 70%-80%. The pulse phase begins by diluting 300 μM 4sU in medium and incubating for 3 h. The chase phase is executed by thawing uridine in medium, resulting in a final concentration of 1 mM. The medium containing 4sU is removed from the cells, followed by two washes with PBS to eliminate any residual 4sU. The medium is then replaced with the prepared uridine-containing medium, initiating the chase period. During the chase phase, the cells are subjected to treatment with or without 500 μM IAA and incubated for 1, 3 and 12 h. To halt the labeling process, 2 ml of TRIzol is added directly to each culture dish. Following thorough lysis, cells were scraped from the culture dish, and the TRIzol-cell mixture was collected in a centrifuge tube, with the addition of 10% *Drosophila* S2 cells (pre-labeled with 100 μM 4sU for 12 h). Then, the 4sU labeled RNA were purified as TT-seq assay without sonication. After final purification, the labeled RNA from each sample was used for constructing transcriptome sequencing libraries using the VAHTS Universal V6 RNA-seq Library Prepkit for Illumina, incorporating various index tags. Following library qualification, samples were pooled based on library concentration and desired data volume, and sequencing was conducted on the Illumina platform using the PE150 sequencing strategy to generate 150-bp paired-end sequencing reads. For 4sU labeling pulse-chase RNA-RT-qPCR, 0.5 μg labeled RNA from each sample was reverse transcribed into cDNA using N9 random and oligo-dT primers, following the instructions provided with HiScript III RT SuperMix for qPCR (Vazyme#R323). Then, qPCR was conducted and analyzed as described in RT-qPCR section (primers see Supplementary Table S7).

### Whole cell lysate-MS and chromatin lysate-MS

For whole cell lysate-MS assay, 1 × 10^6^ cells were washed with PBS and lysed directly in 2 × SDS-loading buffer and heated at 100°C for 15 min. After electrophoresis, the gel was stained with Commassie Blue solution overnight, and destained with 30% ethanol/10% acetic acid in water and prepared for quantitative mass spectrometry (MS) analysis. For chromatin lysate-MS assay, 1 × 10^7^ cells were lysed in ice-cold NP-40 lysis buffer, fractionated on a sucrose cushion, and lysed in glycerol buffer/nuclei lysis buffer to extract the chromatin, similar to the pretreatment in ChIP-seq section. For chromatin fractionation followed by RNase A treatment, the chromatin lysis was incubated with 100 μg/ml RNase A (Takara#2158-1) or 80 U RNasin (Promega#N251B) in 100 μl PBS at 37°C for 1 h. After that, the chromatin lysis was directly in 2 × SDS-loading buffer and heated at 100°C for 15 min. The protein samples were separated on an SDS-PAGE and prepared for quantitative MS analysis, similar to the pretreatment for whole cell lysis-MS assay.

### CUT&tag

1 × 10^6^ mESCs mixed with 10% HEK293T cells were fixed with 0.1% formaldehyde, and they were incubated at room temperature for 2 min. Following this, 0.125 M glycine was added to terminate formaldehyde crosslinking, and the sample were used for CUT&Tag experiment with a commercial kit Hyperactive Universal CUT&Tag Assay Kit for Illumina (Vazyme #TD903). Briefly, after activating conA beads, 95 μl wash buffer was added into samples and incubated for ten min on a rotator at room temperature. Subsequently, anti-H3K9me3 antibodies (Abcam#ab8898), anti-H3K27me3 antibodies (Millipore Sigma#07–449), anti-SUV39H1 antibodies (EMD millipore#05-615) or anti-PCF11 (Abcam#ab134391) was added and incubated overnight at 4°C. Followed by secondary antibody incubation, samples were mixed with pA/G-Tnp for 1 h on rotator. Next, sample was tagmentated in 40 μl tagmentation buffer at 37°C for 1 h. According to the manufacturer's instructions, DNA was extracted and dissolved in 20 μl nuclease-free water. To amplify libraries, add P5/P7 primer (Vazyme#TD202), 2× CAM and perform PCR. PCR clean-up was performed by DNA clean beads and libraries were eluted in 20 μl nuclease-free water and sequencing was conducted using the Illumina platform, employing the PE150 sequencing strategy to generate 150-bp paired-end sequencing reads.

### ChIP-seq and CUT&tag data analysis

The sequenced reads were trimmed using trim_galore (v.0.6.2), and quality control was performed with FastQC (v.0.11.8). Then trimmed reads were mapped to the concatenated index of mouse reference genome (mm10) and human reference genome (hg19) using Bowtie2 (v.2.2.3). The resulting SAM files were converted to BAM files using the SAMtools (v.1.10), after which the BAM files were sorted and indexed, and potential PCR duplicates were removed using sambamba (v.0.7.1). DeepTools (v.3.3.1) was used to visualize the sequencing results. Briefly, two BAM files indicating replicates were merged with samtools merge function, and the merged BAM files were converted into BigWig files using the bamCoverage function. The hg19 reads were calculated as scale factor for spike-in normalization. For RPB1 pSer2/RPB1 and RPB1 pSer5/RPB1 analysis, bigwigCompare function was used by to calculate pSer2 or pSer5 signals over RPB1 signals. For gene tracks, the BigWig files were loaded in Integrative Genomics Viewer (IGV) browser (v.2.11.4). And for meta-gene visualization, the computeMatrix function was used to calculate the occupancy of reads across the specified genome regions, and the plotProfile and plotHeatmap functions were used to plot the data. To identify H3K27me3, H3K9me3, EZH2, KDM6B and SUV39H1 binding genes, Macs2 (v.2.2.7.1) was used to call peaks with the parameters ‘-f BAMPE –keep-dup all –nomodel –bdg –broad’ and peaks with two replicates were merged with IDR (v.2.0.4.2) with the parameter ‘–idr-threshold 0.05’. The final peaks of each protein were intersected with bedtools (v.2.29.2), and the genes with peaks were identified as binding genes.

### Poly(A) RNA-seq data analysis

Reads were trimmed for adapter content with trim_galore (v.0.6.2), and FastQC (v.0.11.8) was used to quality control for trimmed fastq files. The trimmed reads were aligned to concatenated index of mouse genome (mm10) and Drosophila melanogaster genome (dm6) with STAR (v.2.7.3a) with default settings. After selecting the correctly paired and properly mapped reads, as determined by SAMtools (v.1.10) and sambamba (v.0.7.1), featureCounts (v.2.0.1) was used to summarize gene-level read counts per sample from the GENCODE annotation (M23). Differential expression genes were identified with R package DESeq2 (v.1.24.0). mm10 read counts were normalized with size factors estimated on the corresponding reads mapped to the dm6 genome as spike-in normalization. Significant changes in gene expression were determined using a 2-fold cutoff and FDR < 0.05. In order to identify the transcription factors that were enriched in the identified Pol II degradation-upregulated genes, the transcription factor motifs were searched with findMotifsGenome.pl function from Homer (v4.11).

### ChAR-seq and TT-seq data analysis

ChAR-seq and TT-seq mapping and analysis were similar as poly(A) RNA-seq with some modifications. Briefly, paired reads were trimmed with trim_galore (v.0.6.2), and mapped with concatenated mm10-dm6 genome index with STAR (v.2.7.3a). Similar as ChIP-seq, two BAM files indicating replicates were merged with samtools merge function, and the merged BAM files were converted to BigWig files using bamCoverage function, with parameters ‘–filterRNAstrand forward’ or ‘–filterRNAstrand reverse’ to identify sense and anti-sense strand. The dm6 reads was used as spike-in normalization with parameter ‘–scaleFactor’. For strand-specific read counting, featureCounts (v.2.0.1) with parameters ‘-s 2’ was used to count reads distributed in mm10 gene body regions (300 bp downstream of TSS to gene end) and DESeq2 (v.1.24.0) was used to identify differential expression genes with dm6 spike-in normalization. Significant changes in gene expression were determined using a 2-fold cutoff and FDR < 0.05. Deeptools (v.3.3.1) were used to visualize genome distribution and IGV (v.2.11.4) were used to visualize specific gene tracks.

### 4sU pulse-chase data analysis

Sequencing data of 4sU pulse-chase experiments were processed similar as poly(A) RNA-seq. RNA half-life was analyzed as previous reported (32) with some modifications. Briefly, featureCounts was used to summarize gene-level read counts per sample from the GENCODE annotation (M23), and reads mapping to dm6 genome were used as spike-in normalization. The geometric mean of several stable transcripts (*Rplp0*, *Rpsa*, *Rps2*, *Rpl18a*, *Rpl10a*, *Rps5* and *Gapdh*) were used to scaling between samples. Genes with TPM >0.2 in untreated 0 h samples were kept for down-stream analysis. Normalized TPM of each gene was fitted with the first order exponential decay model by using custom R script and RNA half-life was calculated.

### Mass spectrometry (MS) data analysis

Statistical analyses applied to all the proteomic data were performed using the R package DEP (v.1.6.1) with manufacturer's instruction. Briefly, only proteins with no missing values in all replicates of all experimental groups were included subsequent analyses. For chromatin MS data, proteins were searched in uniport database, and nuclear proteins annotated in the subcellular location data based on cell images from SwissBioPics and Gene Ontology (GO) subcellular location annotations were kept for downstream analysis. The significant differences between conditions for each paired comparison were tested, and a cut-off of *P*-value < 0.05 and |Log_2_(Foldchange)| > 1 was used as a filter threshold to classify differentially expressed proteins.

### Pausing, elongation and readthrough index calculation

Pausing and readthrough index were calculate as previous reported with some modifications (33–35). Briefly, pausing index was calculated with RPB1 ChIP-seq data, using the signals of TSSs region (–30 bp to +300 bp of TSSs) divide the signals of gene body region (+300 bp of TSSs region to TESs). Elongation rate was calculated with dividing RPB1 degron TT-seq signals over RPB1 degron RPB1 pSer2 ChIP-seq signals using bigwigCompare function. Readthrough index was calculated with the signals of the forward strand of RPB1 NTD degron TT-seq data, by dividing the average signals in termination windows (2 kb downstream of TESs) by that in pre-termination windows (2 kb upstream of TESs).

### RNA splicing analysis

RNA splicing was analyzed using rMATS as previous reported (36). Briefly, the STAR mapped RNA-seq BAM files were used as input, and parameters ‘-t paired –readLength 150 –cstat 0.0001’ were used. Five alternative splicing events were merged, and the significant differences between conditions for each paired comparison were tested, and a cut-off of FDR < 0.05 and |PSI| > 0.1 was used as a filter threshold to classify splicing changed genes.

### Gene functional enrichment analysis

GO enrichment analysis of chromatin-MS was performed over the differential proteins of interest using the ‘enrichGO’ function of the clusterProfiler R package with a significance cutoff *P* value < 0.05 and *q*-value < 0.1. The Benjamini–Hochberg method was used to correct multiple statistical testing (adjusted *P* values). The gene functional enrichment analysis of Pol II degradation-upregulated genes and upregulated genes identified in UV and α-amanitin treated RNA-seq were performed in Metascape (37) (https://metascape.org/) with specific gene sets.

### Lisa (epigenetic *landscape in silico deletion analysis* and the second descendent of MARGE) analysis

To find potential transcription factors regulating Pol II degradation up-regulated genes, we performed Lisa analysis (38) on http://lisa.cistrome.org/ with the manufacturers’ instruction. Pol II degradation up-regulated genes were set as ‘Gene Set 1’ and short, non-histone genes were set as ‘Backgroud Gene set’ in the hidden options. Transcription factors with RPKM > 1 in RNA-seq were kept for downstream analysis.

## Results

### RNA polymerase depletion decreased RNA metabolic factors and chromatin regulators in chromatin fractions

**Figure 1.**
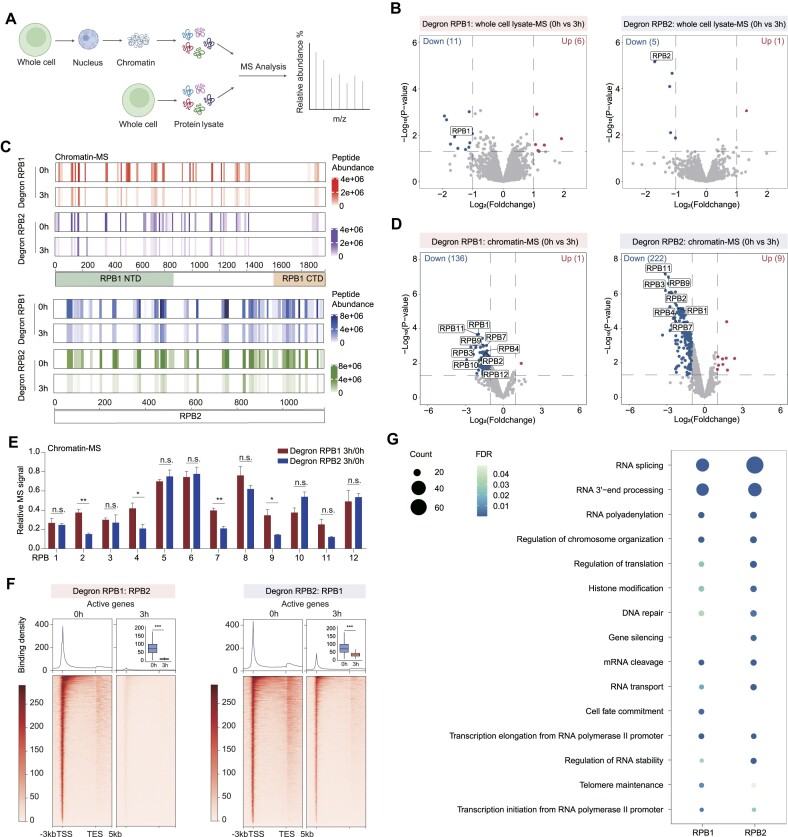
Acute RPB1 and RPB2 degradation induces global Pol II depletion. (**A**) Schematic workflow to identify differential proteins in whole cell lysate-MS and chromatin-MS after RPB1 and RPB2 degradation, respectively. Created with BioRender.com. (**B**) Volcano plots of protein abundance changes from whole cell lysate-MS data of cells with RPB1 (left) or RPB2 (right) depletion, compared with the corresponding untreated cells. Differential proteins were determined with *P*-value < 0.05 and Log_2_(Foldchange) > 1 or Log_2_(Foldchange) < –1. (**C**) Heatmaps indicating peptide abundance of RPB1 (top) and RPB2 (bottom) after RPB1 or RPB2 degradation based on chromatin-MS. Peptide abundance was calculated as the mean value of three replicates. (**D**) Volcano plots of protein abundance changes from chromatin lysate-MS data of cells with RPB1 (left) or RPB2 (right) depletion, compared with the corresponding untreated cells. Differential proteins were determined with *P*-value < 0.05 and Log_2_(Foldchange) > 1 or Log_2_(Foldchange) < –1. (**E**) Bar plot indicating protein abundance changes of 12 Pol II subunits in chromatin-MS after RPB1 and RPB2 degradation, respectively. Protein abundance of 3h degradation was normalized to 0 h data. The values were plotted as the means ± SEMs. Statistical significance was determined by a two-tailed *t* test with 3 replicates. n.s. not significant. *P < 0.05, **P < 0.01. (**F**) Metagene profiles of normalized RPB2 and RPB1 ChIP-seq reads after RPB1 or RPB2 degradation in active genes (*n* = 9077), respectively. Active genes were defined as genes with RPKM > 1 in TT-seq. The box plots showed the comparison of changes in the transcription start sites (TSSs). Statistical significance was assessed by a two-sided Wilcoxon test. ***P < 0.001. (**G**) Gene functional enrichment analysis of differential proteins after RPB1 or RPB2 degradation in chromatin-MS. The size of circles indicated protein counts of each term, and the colors indicated FDR.

We leveraged the previously established RNA polymerase subunit degradation system in mESCs to uncover molecular insights into global Pol II transcription repression (29,30). A 3-h auxin treatment targeted the subunit for degradation, following which whole cell extracts and chromatin fractions underwent mass spectrometry analysis post RPB1 or RPB2 depletion (Figure 1A, Supplementary Table S1). Volcano plot analyses of whole cell extracts revealed minimal changes in protein abundance outside the targeted proteins, indicating the specificity of the auxin-inducible degron technique (Figure 1B). Mass spectrometry analyses of chromatin fractions primarily indicated specific degradation of RPB1 or RPB2, and decreased chromatin occupancy of protein factors (*n* = 136 for RPB1 and *n* = 222 for RPB2) (Figures 1C, D, Supplementary Figure S1A, B), suggesting that Pol II is essential for the assembly of many protein factors at chromatin.

To gain comprehensive molecular insights following RPB1 or RPB2 depletion, we conducted further analysis of chromatin mass spectrometry data and performed ChIP-seq analyses (Supplementary Table S2). The protein abundance for all 12 subunits of Pol II in chromatin fractions displayed a decrease in their levels (Figure 1E). Notably, RPB2 depletion showed relatively more decreased abundance for RPB4, RPB7, RPB9 and RPB11 (Figure 1E), suggesting these subunits are more reliant on RPB2 compared to RPB1, aligning with the 3D crystal structures of the Pol II complex. Both RPB1 depletion followed by RPB2 ChIP-seq and RPB2 depletion followed by RPB1 ChIP-seq validated the mass spectrometry data and corresponded to the disassembly of the Pol II complex at chromatin (Figures 1F). The decrease in RPB2 ChIP-seq signals was more pronounced compared to RPB1 ChIP signals. However, the reduction in RPB2 protein abundance after RPB1 depletion is not as dramatic as the decrease in RPB1 protein abundance following RPB2 depletion in mass spectrometry. This inconsistency may reflect variations in different techniques.

RNA polymerase II is believed to be intricately linked with various RNA and DNA metabolic processes in nucleus yet systematic investigations have been lacking. We conducted GO function analyses for the proteins displaying decreased abundance in chromatin fractions after RNA polymerase depletion (Figure 1G). The results indicated enrichment of RNA processing factors, including RNA splicing, RNA cleavage, RNA polyadenylation, RNA stability, RNA export, and translation, suggesting that RNA polymerases may coordinate RNA fate determination from transcription to translation. These results were consistent with previous reports by Choder and colleagues involves the Pol II subunits Rpb4 and Rpb7, which have been implicated in mRNA decay and translation in yeast (9,39). Additionally, the decreased protein abundance proteins were also enriched in histone modification and chromosome organization, implying an intimate connection between chromatin and RNA polymerase II. Furthermore, certain functional categories such as cell fate commitment and telomere maintenance merit exploration in future studies. This mass spectrometry dataset will serve as a valuable resource for candidate protein factors that functionally crosstalk with RNA polymerase II at chromatin in mammalian cells.

Given that Pol II degradation may lead to decreased levels of nascent RNAs and their associated proteins on chromatin, we undertook a detailed exploration of protein categories. This involved performing chromatin fractionation followed by RNase A treatment to isolate RNA-mediated chromatin-associated proteins. Our analysis revealed a decrease in subsets of proteins (*n* = 89) in the chromatin preparations, while we also noted an increase in the abundance of many proteins (*n* = 111) in the chromatin fractions, suggesting that RNA may suppress the recruitment of certain chromatin regulators, as previously reported (40). Through overlapping analyses of proteins downregulated by RNase A treatment and those affected by RPB1 and RPB2 depletion in the chromatin fractions, we identified several RNA binding proteins (Supplementary Figure S1C, D). However, we observed that many proteins downregulated by RPB1 or RPB2 depletion did not overlap with those affected by RNase A treatment, indicating that Pol II-associated nascent RNA contributes to the protein composition associated with chromatin, alongside the critical role played by Pol II subunits themselves in this process. These results are in line with recent discoveries of direct interactions between spliceosome components and Pol II (41), as well as the concept of liquid-liquid phase separation of Pol II CTD with gene expression regulators (42,43). Additionally, we conducted an overlap analysis between proteins dissociating from chromatin post-RPB1 and RPB2 degradation and our previously published RPB1 and RPB2 ChIP-MS data (30). Despite potential technical variations between the ChIP-MS and chromatin preparation mass spectrometry experiments conducted by different individuals at different times, we found that some of the overlapping proteins are known to participate in RNA transcription processes (Supplementary Figure S1E–G). Collectively, these findings suggest that the degradation of Pol II RPB1 or RPB2 subunits can directly and indirectly lead to loss of nascent RNA and transcription-associated RNA-binding proteins with chromatin. This underscores the complex interplay between transcription, RNA processing, and chromatin dynamics in orchestrating gene expression.

### The degradation of RNA polymerase resulted in increased gene expression for certain gene subsets

**Figure 2.**
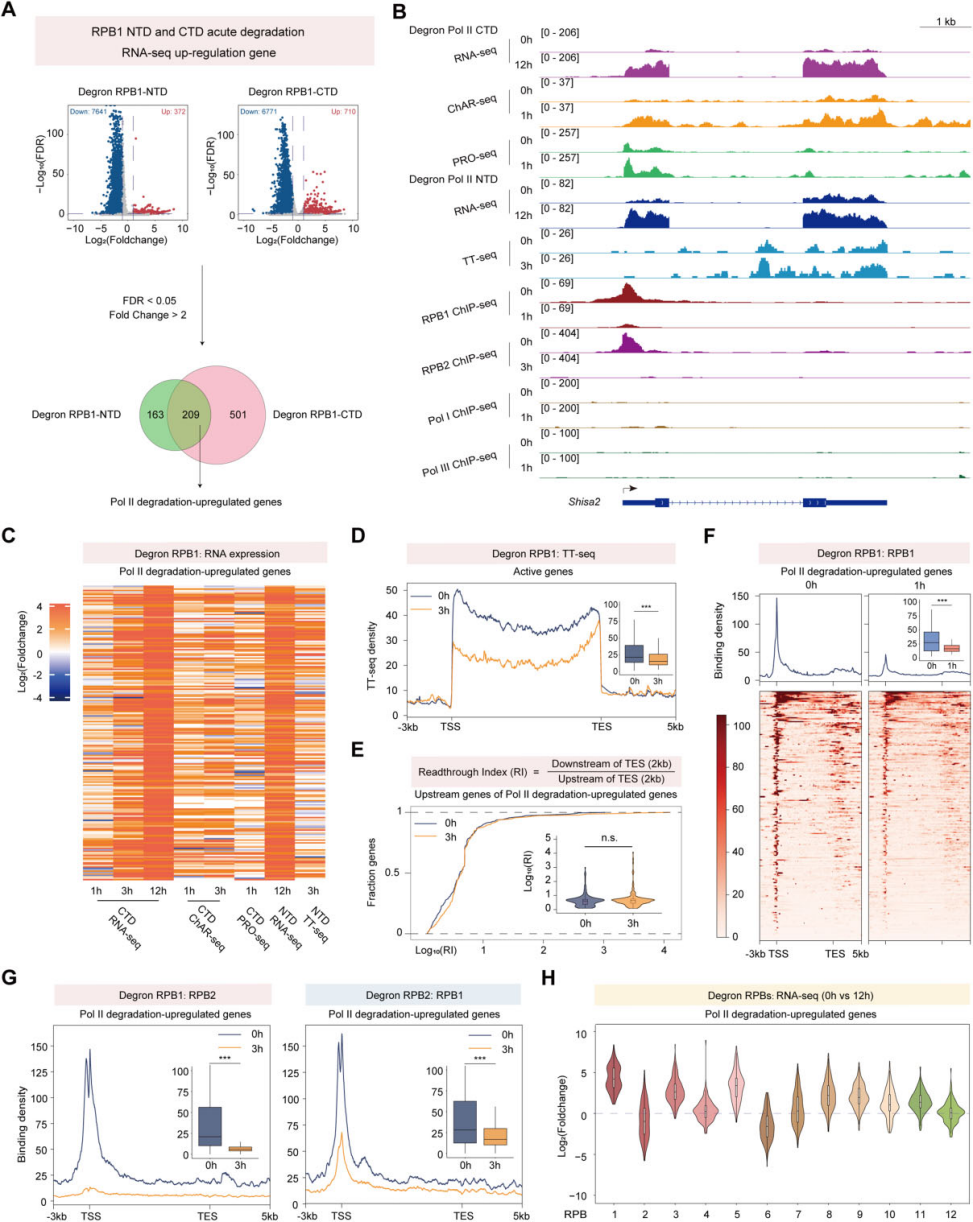
RPB1 degradation resulted in increased gene expression for subsets of genes. (**A**) Schematic workflow to identify Pol II degradation-upregulated genes. Pol II degradation-upregulated genes were overlap upregulated genes in the RPB1 NTD and CTD degradation RNA-seq with RPKM > 1. (**B**)Tracks for *Shisa2* which is a Pol II degradation-upregulated gene of different sequencing data after Pol II CTD or NTD degradation for the indicated durations. (**C**) Heatmap showing Log_2_(Foldchange) of Pol II degradation-upregulated genes in different sequencing data. (**D**) Metagene profiles of normalized TT-seq reads after RPB1 degradation in active genes. The box plots showed the comparison of changes in the gene body regions. Statistical significance was assessed by a two-sided Wilcoxon test. ***P < 0.001. (**E**) Empirical cumulative density function (ECDF) and violin plots showing the changes in the readthrough index of upstream genes of Pol II degradation-upregulated genes induced by Pol II NTD degradation based on TT-seq signals. The upstream genes were extracted as the nearest upstream genes of Pol II degradation-upregulated genes according to GENCODE annotation (M23). Statistical significance was assessed using a two-sided Wilcoxon test. n.s. not significant. (**F**) Metagene profiles of normalized Pol II ChIP-seq reads after RPB1 degradation in Pol II degradation-upregulated genes. The box plots showed the comparison of changes in TSSs. Statistical significance was assessed by a two-sided Wilcoxon test. ***P < 0.001. (**G**) Metagene profiles of normalized RPB2 and RPB1 ChIP-seq reads after RPB1 or RPB2 degradation in Pol II degradation-upregulated genes, respectively. The box plots showed the comparison of changes in the transcription start sites (TSSs). Statistical significance was assessed by a two-sided Wilcoxon test. ***P < 0.001. (**H**) Violin plot showing Log_2_(Foldchange) of Pol II degradation-upregulated genes after 12 Pol II subunits degradation.

We subsequently conducted spike-in RNA-seq analyses using the C-terminal degron and N-terminal degron of RPB1 in mESCs, which we previously constructed (35). Previous works have demonstrated that the CTD degron of RPB1 exclusively degrades its CTD, whereas the NTD degron of RPB1 degrades the full-length RPB1 (23,35). Both scenarios result in the downregulation of gene expression. Surprisingly, a subset of genes displayed increased RNA-seq signals. Overlapping analyses of RPB1 CTD and NTD degron revealed 209 genes (defined as Pol II degradation regulated genes) with upregulated mature RNA levels (Figure 2A, Supplementary Tables S3 and S4). Individual gene analysis demonstrated that total Pol II levels decreased, accompanied by increases in mature, chromatin-associated, and nascent RNAs after RPB1 degradation within the *Shisa2* gene locus. Conversely, Pol I and Pol III occupancy at the upregulated gene remained unchanged (Figure 2B). For simplicity, we employed the RPB1-NTD degron (referred to as RPB1) in the subsequent analyses of this study.

To eliminate potential secondary effects or transcriptional compensation following Pol II depletion, we analyzed previously published mature RNA, chromatin-associated RNA, and nascent RNA immediately after RPB1 depletion at different time points (29–30,44). We consistently observed upregulation of these genes, further validated by RT-qPCR (Figures 2C, Supplementary Figures S1H, I, S2A). Subsequently, following RPB1 and RPB2 degradation, a TT-seq dataset was generated and revealed a roughly similar trend for subset gene activation and nearly complete repression after RPB1 and RPB2 degradation, respectively (Figures 2C, D, Supplementary Figure S2B–L). The upregulation across different datasets generally exhibits similarity and correlation (Figures 2C, Supplementary Figure S2B–L), although some variations are observed, possibly attributable to experimental differences. Given previous findings that degradation of the CTD of RPB1 leads to transcriptional readthrough (23), which may lead to upregulations of their downstream genes, we calculated the readthrough index for upstream genes for these Pol II degradation-upregulated genes and found no change (Figure 2E). Additionally, RPB1 and RPB2 occupancy at these upregulated genes decreased, indicating a reduction in total Pol II at these sites (Figures 2F, G). Further analysis of published RNA-seq data following the degradation of 12 Pol II subunits revealed that most other Pol II subunit depletions also resulted in upregulation of these genes (Figure 2H, Supplementary Figure S2M, N, Supplementary Table S5) (30). However, minimal changes were observed after RPB4 and RPB12 depletion, possibly attributable to splicing isoforms as previously suggested (30). Surprisingly, RPB2 and RPB6 depletion caused a decrease in the expression of these genes, suggesting that these two subunits may have a more potent effect on transcriptional inhibition than RPB1. These observations are anticipated, given that RPB2 serves as the second largest subunit of Pol II, and RPB6 is essential for Pol II assembly. Consequently, we employed RPB2 as a control for our subsequent analyses.

### Pol II degradation upregulated genes are enriched in GPCR ligand binding, polycomb bound, less paused short genes

**Figure 3.**
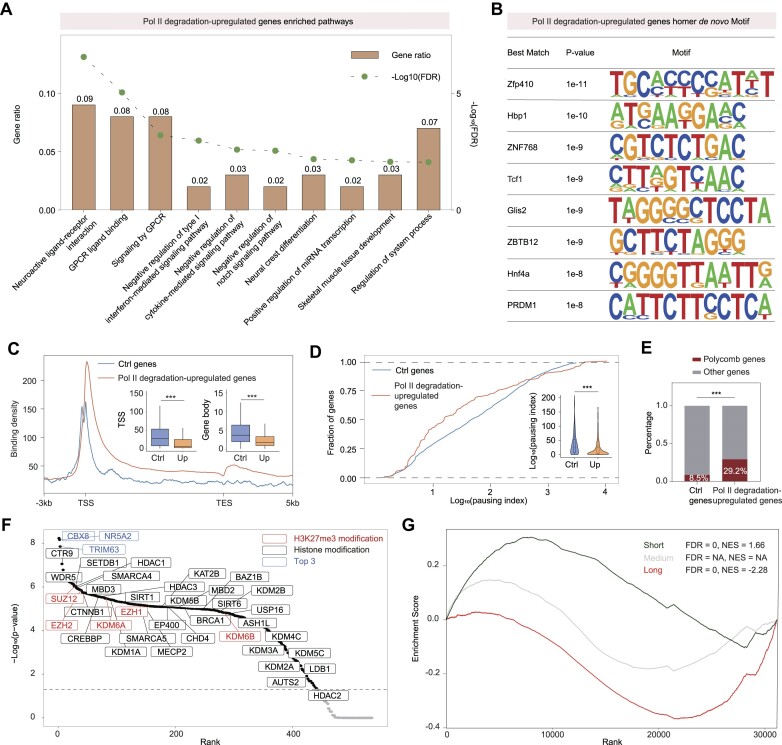
Pol II degradation-upregulated were enriched in GPCR ligand binding, polycomb bound and less paused short genes. (**A**) Gene functional analysis of Pol II degradation-upregulated genes. The bar plot indicated the gene ratio of each term, and the scatter on each bar indicated the –Log_10_(FDR). (**B**) List of transcription factor motifs that were enriched in Pol II degradation-upregulated genes, and the corresponding p-values (which are calculated using HOMER with cumulative binomial distributions) is shown. (**C**) Metagene profiles of Pol II ChIP-seq reads in ctrl genes (length < 10 kb, without histone genes and non-upregulated) and Pol II degradation-upregulated genes. The box plots showed the comparison of ctrl genes and Pol II degradation-upregulated genes in TSSs (left) and gene body regions (right). Statistical significance was assessed by a two-sided Wilcoxon test. ***P < 0.001. (**D**) ECDF and violin plots showing the changes in the pausing index of ctrl genes and Pol II degradation-upregulated genes based on Pol II ChIP-seq signals. Statistical significance was assessed using a two-sided Wilcoxon test. ***P < 0.001. (**E**) Bar plot showing the ratio of polycomb genes in Pol II degradation-upregulated genes and ctrl genes. Statistical significance was assessed using Pearson's Chi-squared test. ***P < 0.001. (**F**) Lisa analysis identifying potential regulators for Pol II degradation-upregulated genes with setting ctrl genes as background in ‘Background Gene Set’ options in http://lisa.cistrome.org/. The y axis indicating the –Log_10_(*P*-value) of the most relevant samples of indicated transcription factors and the x axis indicating the ranking of enriched transcription factors. The grey dashed line showed the cut off of *P*-value = 0.05. H3K27me3 modification related, histone modification related and the top3 indicated regulators were indicated as red, black and blue, respectively. (**G**) Gene set enrichment analysis (GSEA) of RNA-seq data after Pol II CTD degradation. All genes were classified into 3 groups: Short (0–10 kb), Medium (10–75 kb) and Long (>75 kb) according to GENCODE annotation (M23). The x axis indicating the ranking of Log_2_(Foldchange) in RNA-seq data, and the y axis indicating the enrichment score. The FDR and NES in the top right-hand corner indicating the false discovery rate and normalized enrichment score, respectively. NA indicated not significant. Up regulated genes were enriched in the Short group, and down regulated genes were enriched in the Long group. The Medium group is not significant.

We then conducted characterization analyses for the genes upregulated following Pol II degradation. Gene functional enrichment analyses revealed enrichment in GPCR ligand binding, signaling and development, suggesting their potential role in aiding cells to overcome transcriptional shutdown under various environmental stimuli (Figure 3A). The upregulation of G protein-coupled receptor (GPCR) signaling following RPB1 degradation may represent a cellular response aimed at adapting to compromised transcriptional machinery. GPCRs, with their diverse signaling cascades, can activate downstream effectors that aid cells in adapting to stress conditions (45,46). For example, they can influence intracellular cAMP levels, activate protein kinase A (PKA), and trigger various signaling pathways leading to changes in gene expression, metabolic adjustments, and cellular protective mechanisms (47–49). This suggests that these genes could play a role in modulating cellular responses to environmental cues, stress, or other signaling pathways. Concerning the potential impact of RPB1 deletion on the neuroactive ligand-receptor pathway, the upregulation of GPCR genes involved in neuroactive ligand-receptor interactions might be an attempt to enhance or modify neuronal signaling pathways to adjust to the altered transcriptional environment for differentiation (50–52).

Motif analysis of the promoter sequences of these upregulated genes showed enrichment for specific transcription factors involved in differentiation or regulators of specific signaling pathways, such as ZFP410, HBP1, TCF1, GLIS2, HNF4A and PRDM1 (Figure 3B) (53–58). Further exploration of the regulation of these transcription factors may provide insights into the transcriptional changes following Pol II degradation. In a prior study, resistant Pol II was found to be enriched for less paused Pol II after Triptolide-induced transcription inhibition (59). We subsequently examined the Pol II occupancy for genes upregulated following Pol II degradation. Both meta-gene and pausing index analyses indicated that these genes are enriched in less paused Pol II and exhibit low expression levels in untreated mESCs (Figures 3C, D). Additionally, we analyzed existing ChIP-seq signals for these upregulated genes and observed enrichment in Polycomb regulators, including methyltransferases and demethylases of H3K27me3. These genes are relatively enriched among previously identified Polycomb targets (Figures 3E, F) (60). Notably, a previous study under UV treatment demonstrated downregulation of long active genes and increased expression of short genes associated with cancer (24,61). Our Gene Set Enrichment Analysis (GSEA) revealed that Pol II degradation upregulated genes are enriched in short genes (<10 kb) (Figure 3G). These findings suggest that these upregulated genes may not merely represent random compensation for transcriptional shutdown, but rather serve specific functions and could be regulated by distinct transcriptional and epigenetic mechanisms.

### RPB1 degradation correlates with H3K27me3 demethylation, while RPB2 correlates with H3K9me3 methylation

**Figure 4.**
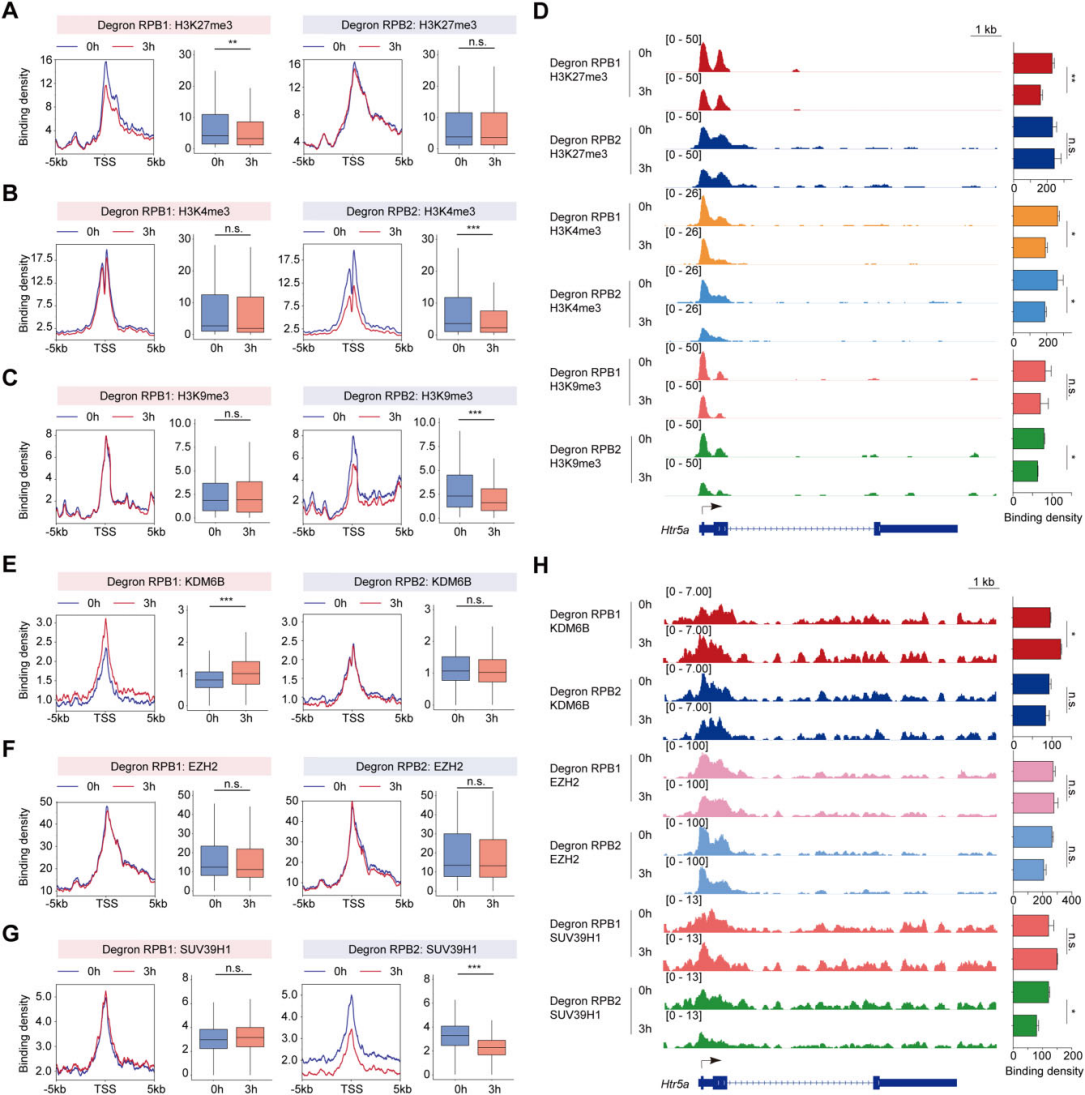
RPB1 degradation resulted in decreased H3K27me3 occupancy, while RPB2 degradation led to decreased H3K9me3. (**A–C**) Metagene profiles of normalized binding density of H3K27me3, H3K4me3 and H3K9me3 at the TSSs of Pol II degradation-upregulated genes after RPB1 (left) and RPB2 (right) degradation, respectively. The box plots showed the comparison of changes in the TSSs. Statistical significance was assessed by a two-sided Wilcoxon test. n.s. not significant. **P < 0.01, ***P < 0.001. (**D**) Tracks (left) for *Htr5a* which is a Pol II degradation-upregulated gene of H3K27me3, H3K4me3 and H3K9me3 binding after RPB1 and RPB2 degradation, respectively. The bar plot (right) showed the comparison of changes in the –3 kb to +3 kb of TSSs for indicated track data. The values were plotted as the means ± SEMs. Statistical significance was determined by a two-tailed *t* test with two replicates. n.s. not significant. *P < 0.05, **P < 0.01. (**E–G**) Metagene profiles of normalized binding density of KDM6B, EZH2 and SUV39H1 at the TSSs of Pol II degradation-upregulated genes after RPB1 (left) and RPB2 (right) degradation, respectively. The box plots showed the comparison of changes in the TSSs. Statistical significance was assessed by a two-sided Wilcoxon test. n.s. not significant. ***P < 0.001. (**H**) Tracks (left) for *Htr5a* which is a Pol II degradation-upregulated gene of KDM6B, EZH2 and SUV39H1 binding after RPB1 and RPB2 degradation, respectively. The bar plot (right) showed the comparison of changes in the –3 kb to +3 kb of TSSs for indicated track data. The values were plotted as the means ± SEMs. Statistical significance was determined by a two-tailed *t* test with two replicates. n.s. not significant. n.s. not significant. *P < 0.05

To explore the relationship between Pol II degradation upregulated genes and epigenetic regulation, we conducted chromatin binding assays for active marker H3K4me3, poised marker H3K27me3, and silent marker H3K9me3 after depletion of RPB1 and RPB2. Meta and single gene analyses revealed that RPB1 depletion led to decreased H3K27me3 levels without affecting H3K4me3 and H3K9me3, while RPB2 depletion resulted in reduced H3K4me3 and H3K9me3, with no change in H3K27me3, both for upregulated genes (Figures 4A–D) and in global analyses (Supplementary Figures S3A–C). The greater the transcription activation following RPB1 degradation, the more pronounced the H3K27me3 modifications for the genes, and the greater the reduction in H3K27me3 levels after RPB1 depletion (Supplementary Figure S3D). H3K4me3 levels remained relatively stable following RPB1 degradation, contrasting with the decrease observed after RPB2 depletion at transcription start sites for genes up-regulated by Pol II (RPB1) degradation. These up-regulated genes exhibited reduced transcription post-RPB2 depletion, aligning with the decrease in H3K4me3 levels. However, despite the stability of H3K4me3 at the promoter regions of these genes following RPB1 degradation, this observation likely reflects combined effects arising from RPB1 depletion, or the accessibility of the promoters of these genes, which may not have required remodeling of their histone modifications.

To delve deeper, we performed chromatin binding analyses for the H3K27me3 writer EZH2, the eraser KDM6B, and the H3K9me3 writer SUV39H1 using high-quality antibodies that were available. Consistent with the observed histone modification changes, we noted an increase in KDM6B following RPB1 depletion and a decrease in SUV39H1 after RPB2 depletion, both in single gene and global analyses for the upregulated genes (Figures 4E–H, Supplementary Figure S3E–G). These results suggest that while the investigated epigenetic changes may not directly explain the upregulation of genes due to Pol II depletion, they do indicate that RPB1 is associated with H3K27me3 demethylation, and RPB2 facilitates H3K9me3 modification. This aligns with previous findings indicating that RPB2 is necessary for heterochromatin formation in yeast (62).

### RPB1 degradation reduces pSer2 from most active genes, while increasing pSer2 for RPB1 depletion upregulated genes

**Figure 5.**
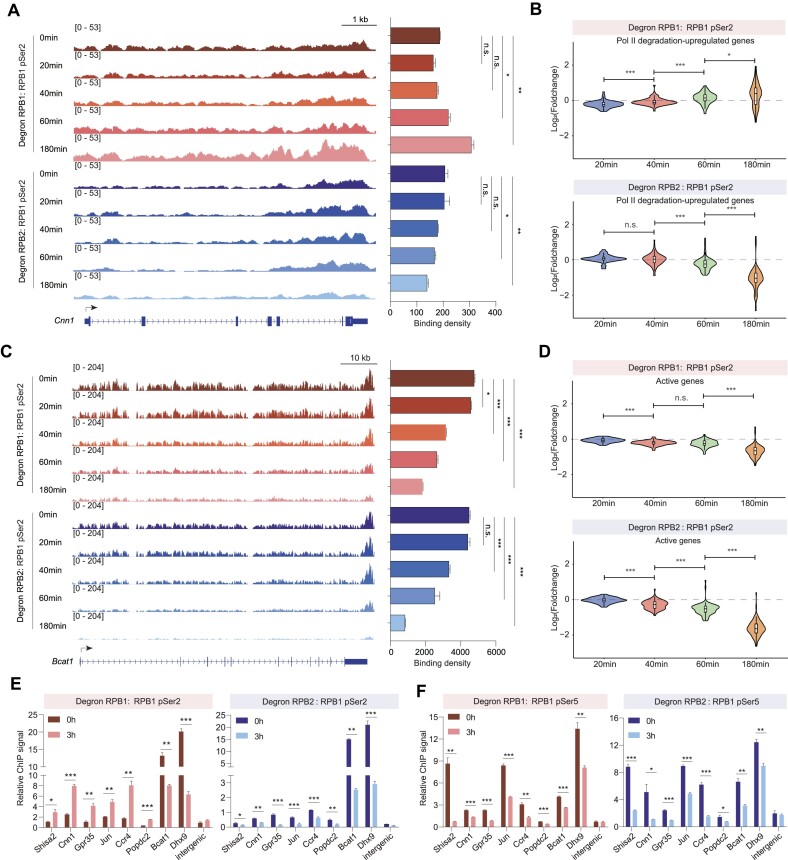
RPB1 degradation resulted in increased pSer2 occupancy in Pol II degradation-upregulated genes. (**A**) Tracks (left) for *Cnn1* which is a Pol II degradation-upregulated gene of time-course pSer2 ChIP-seq after RPB1 (top) and RPB2 (bottom) degradation. The bar plot (right) showed the comparison of changes in the gene body regions for indicated track data. The values were plotted as the means ± SEMs. Statistical significance was determined by a two-tailed *t* test with two replicates. n.s. not significant. *P < 0.05, **P < 0.01. (**B**) Violin plot showing Log_2_(Foldchange) of normalized pSer2 ChIP-seq reads of Pol II degradation-upregulated genes in indicated time point compared with 0min. Statistical significance was assessed by a two-sided Wilcoxon test. n.s. not significant. *P < 0.05, **P < 0.01, ***P < 0.001. (**C**) Tracks (left) for *Bcat1* which is an active gene of time-course pSer2 ChIP-seq after RPB1 (top) and RPB2 (bottom) degradation. The bar plot (right) showed the comparison of changes in the gene body regions for indicated track data. The values were plotted as the means ± SEMs. Statistical significance was determined by a two-tailed *t* test with two replicates. n.s. not significant. *P < 0.05, ***P < 0.001. (**D**) Violin plot showing Log_2_(Foldchange) of normalized pSer2 ChIP-seq reads of active genes in indicated time point compared with 0min. Statistical significance was assessed by a two-sided Wilcoxon test. n.s. not significant, ***P < 0.001. (**E**) RPB1 pSer2 ChIP-qPCR analysis of gene body regions of selected Pol II degradation-upregulated genes after degradation of RPB1 (left) and RPB2 (right) for 3 h. *Bcat1* and *Dhx9* were active genes control. The values were normalized to input and plotted as the means ± SEMs. Statistical significance was determined by a two-tailed *t* test with at least three replicates. *P < 0.05, **P < 0.01, ***P < 0.001. (**F**) Similar as (E) RPB1 pSer5 ChIP-qPCR analysis of TSSs of selected Pol II degradation-upregulated genes after degradation of RPB1 (left) and RPB2 (right) for 3 h. *Bcat1* and *Dhx9* were active genes control. The values were normalized to input and plotted as the means ± SEMs. Statistical significance was determined by a two-tailed *t* test with at least three replicates. *P < 0.05, **P < 0.01, ***P < 0.001.

Despite the decrease in total Pol II for the Pol II degradation upregulated genes, we conducted serine 2 phosphorylated Pol II ChIP-seq after RPB1 and RPB2 depletion at different time points. The pSer2 ChIP-seq results indicated an increase after RPB1 deletion, while pSer2 continued to decrease after RPB2 depletion for the Pol II degradation upregulated genes (Figures 5A, B). Both single and global analyses for active genes revealed decreased pSer2 following RPB1 and RPB2 depletion (Figures 5C, D, Supplementary Figure S4A–E). These findings align with RNA-seq expression data, suggesting that pSer2 at these upregulated genes are resistant or even increased after RPB1 depletion, while it is prone to degradation after RPB2 depletion. Additionally, the increase in pSer2 associated with genes upregulated after RPB1 degradation was validated via ChIP-qPCR, while a decrease in pSer5 was observed for these genes (Figures 5E, F). These results imply that serine 2 phosphorylated and unphosphorylated Pol II respond differently to RPB1 degradation but similarly to RPB2 depletion.

We conducted analyses on the Ser2P/Pol II and Ser5P/Pol II ratios to investigate whether the changes in Ser2 (or Ser5) levels were due to variations in total Pol II signal or alterations in Ser2 and Ser5 phosphorylation levels (Supplementary Figures S5A–E). Our results indicated an elevation in Ser2P/Pol II and Ser5P/Pol II levels for upregulated and active genes post-RPB1 depletion, underscoring the dominant role of Pol II decrease as a phenotype. Conversely, after RPB2 depletion, we observed no significant shifts in Ser2P/Pol II and Ser5P/Pol II ratios for upregulated genes; however, there was a decrease in the Ser2P/Pol II ratio and a slight increase in the Ser5P/Pol II ratio for active genes following RPB2 degradation. This suggests that the effects of RPB2-induced Pol II degradation differ from those of RPB1 depletion concerning both total and phosphorylated Pol II. We realized that an elevation in Pol II signal, particularly Ser2 phosphorylated Pol II (Ser2P), may stem from an increased number of transcribing Pol II and/or a reduction in elongation rate. We calculated the elongation rate by dividing the TT-seq signals by the Ser2P occupancy signals and found no significant alterations pre- and post-depletion of RPB1 for both the up-regulated genes and active genes (Supplementary Figure S6A). This outcome aligns with the increase in elongating Pol II, indicating stability in the elongation process.

### Elevated RNA half-life contributes to the upregulation of specific genes following RPB1 depletion

**Figure 6.**
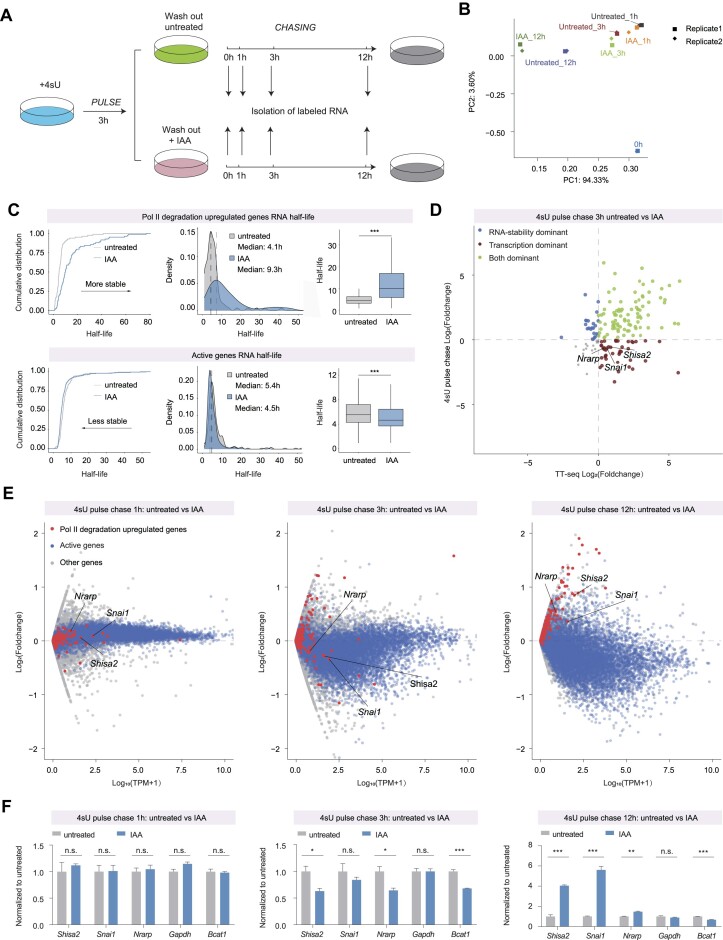
Elevated RNA half-life contributes to the upregulation of specific genes following RPB1 depletion. (**A**) Schematic workflow of 4sU pulse chase experiments. (**B**) Principal component analysis (PCA) of read counts in different time point after 4sU wash out in untreated and RPB1 degradation cells. (**C**) Empirical cumulative density function (ECDF) (left), density plot (middle) and violin plot (right) indicating RNA half-life of Pol II degradation-upregulated genes (top) and active genes (bottom) before and after RPB1 degradation. Statistical significance was assessed using a two-sided Wilcoxon test. ***P < 0.001. (**D**) Scatter plot showing the Log_2_(Foldchange) of TT-seq and 4sU pulse chase RNA-seq of Pol II degradation-upregulated genes before and after RPB1 degradation (3h). Green, TT-seq > 0 and 4sU pulse chase > 0, blue, TT-seq < 0 and 4sU pulse chase > 0, red, TT-seq > 0 and 4sU pulse chase < 0. Note: some Pol II degradation-regulated genes were identified by RNA-seq, but showed decreased TT-seq signals, which may reflect the variations between different techniques. (**E**) MA-plot showing the mean expression and Log_2_(Foldchange) of Pol II degradation-upregulated genes (red), and active genes (blue) before (0 h) and after 1 h (Left), 3 h (Middle) and 12 h (Right) RPB1 degradation, respectively. (**F**) RT-qPCR of selected Pol II degradation-upregulated genes (*Shisa2*, *Nrarp*, *Snai1*), stable gene (*Gapdh*) and active gene (*Bcat1*) from 4sU pause chase labeled RNA after RPB1 degradation. The values were firstly normalized to Drosophila_CG10433 transcript expression, and then normalized to the values of 0 h. Bar plots were shown as means ± SEMs. Statistical significance was determined by a two-tailed *t* test with at least three replicates. n.s. not significant. *P < 0.05, **P < 0.01, ***P < 0.001.

To delve specifically into quantifying the impact of RNA stability on these up-regulated genes, we conducted a 4sU labeling pulse-chase RNA-seq experiment following RPB1 depletion at various time points (0, 1, 3 and 12 h). These data exhibit high reproducibility and demonstrate a gradual decline in RNA-seq signals over increased depletion durations, indicating the efficacy of our experiments (Figures 6A–C, Supplementary Table S6). Subsequently, we calculated RNA half-lives utilizing a previously published method (32,63). Our findings reveal a significant increase in RNA half-life for RPB1 depletion up-regulated genes and a slight decrease for active genes. Through a comparative analysis between TT-seq (reflecting transcriptional levels) and RNA half-life changes (representing post-transcriptional processes), we noticed that while some upregulated genes exhibited enhanced RNA half-life, subsets of genes displayed decreased RNA half-life but increased TT-seq signals after 3h of RPB1 depletion, suggesting upregulation due to heightened transcription activity (Figure 6D). Notably, model genes like *Shisa2*, *Snail1* and *Nrarp* exemplify this pattern (Figure 6E). However, after 12 h of RPB1 depletion, a majority of up-regulated genes demonstrated increased RNA half-life, posing challenges in discerning whether this change derives from secondary effects post-transcriptional inhibition. These results could benefit from further validation through 4sU pulse-chase RT-qPCR experiments (Figure 6F). Collectively, our findings suggest that although increased RNA half-life plays a role in the upregulation of specific genes following RPB1 depletion, enhanced transcription also emerges as a factor explaining the upregulation of certain genes rather than relying solely on changes in RNA half-life after Pol II degradation for 3 h.

Given PCF11’s pivotal role in transcription termination in mammalian cells, we conducted RPB1 degradation followed by PCF11 Cut&Tag analyses. Our findings revealed that PCF11 exhibited enhanced localization at the Transcription End Sites (TESs) regions in genes upregulated by Pol II degradation as well as in actively transcribed genes (Supplementary Figure S6B). The observed effects of PCF11-mediated transcription termination do not align with the upregulation of genes affected by Pol II degradation. Analysis of read-through signals post-TESs did not show noticeable alterations following RPB1 depletion (Supplementary Figure S6C). Moreover, our investigation into genes undergoing altered RNA splicing revealed no overlap when compared with the set of genes upregulated by Pol II degradation (Supplementary Figure S6D). Furthermore, RT-qPCR analyses on model genes (*Shisa2*, *Snai1* and *Nrarp*) across cytoplasmic and nuclear fractions pre- and post-RPB1 depletion indicated no significant distribution changes for the upregulated genes influenced by Pol II degradation for 3h (Supplementary Figures S6E,F). We did observe a decreased nuclear/cytoplasm ratio after 12 h of Pol II depletion, a change that could potentially be attributed to the extended RNA half-life. These collective outcomes suggest that mechanisms involving PCF11-mediated transcription termination, mRNA splicing, 3′ processing, and RNA export do not directly account for the immediate upregulation seen in genes influenced by Pol II degradation.

### Alpha-amanitin or UV treatment caused the RPB1 degradation, global transcription repression and activation of a subset of genes

**Figure 7.**
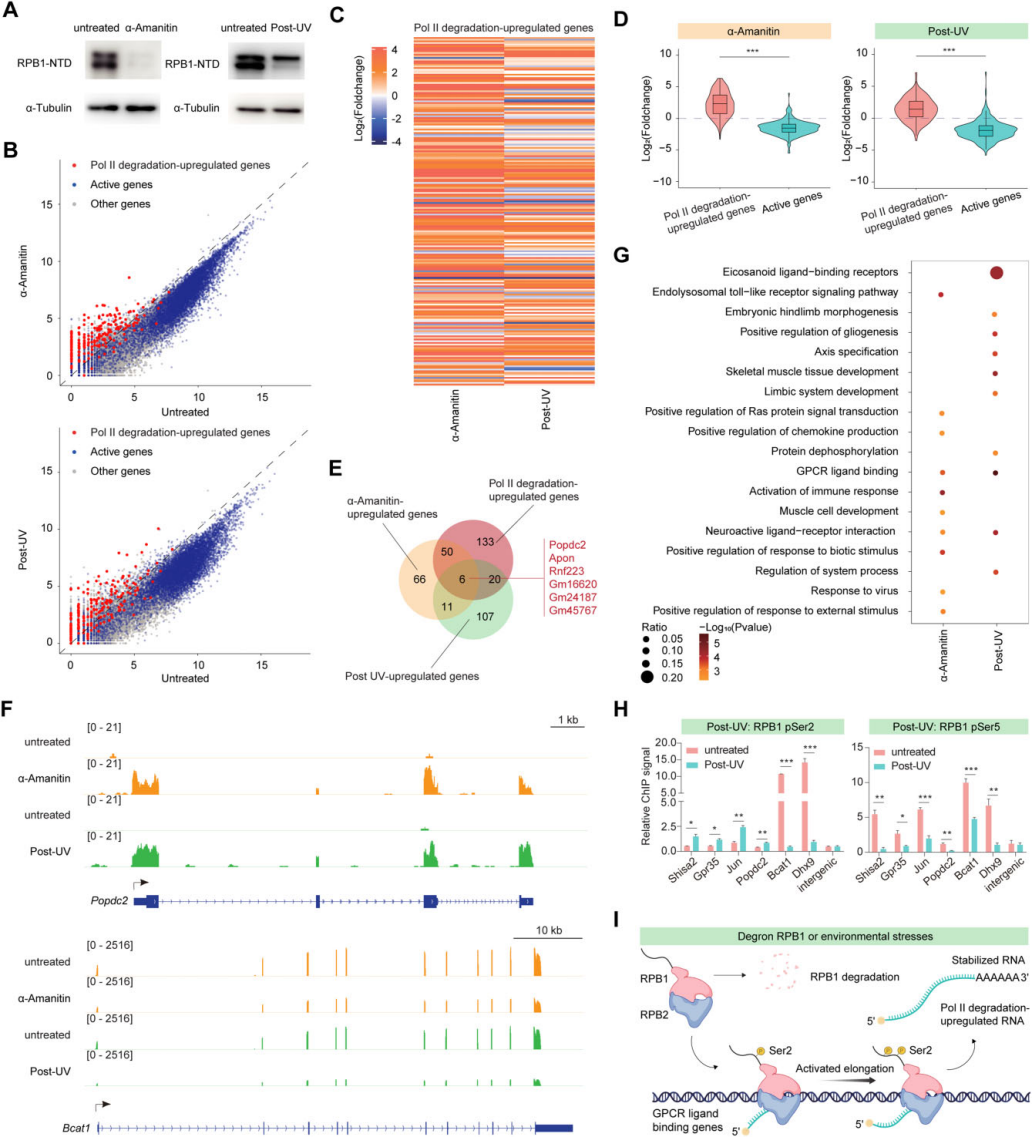
α-Amanitin and UV treatment caused the degradation of RPB1 and activated the expression of subsets of genes. (**A**) Western blot analysis of RPB1 after α-amanitin treatment for 24 h (left), and UV irradiation with 3 h recovery (right). (**B**) Scatterplots showing gene expression changes after α-amanitin treatment for 24 h (top) and UV irradiation with 12 h recovery (bottom). Blue points indicated active genes, and red points indicated Pol II degradation-upregulated genes. (**C**) Heatmap showing the Log_2_(Foldchange) of Pol II degradation-upregulated genes after α-amanitin treatment for 24 h (left) and UV irradiation with 12 h recovery (right). (**D**) Violin plot showing the Log_2_(Foldchange) of Pol II degradation-upregulated genes after α-amanitin treatment for 24 h (left) and UV irradiation with 12 h recovery (right). Statistical significance was assessed by a two-sided Wilcoxon test. ***P < 0.001. (**E**) Venn plot showing the overlap of up-regulated genes after α-amanitin treatment for 24 h and UV irradiation with 12 h recovery with *P*-value < 0.05 and Log_2_(Foldchange) > 1, and Pol II degradation-upregulated genes. (**F**) Tracks for *Popdc2* (top) and *Bcat1* (bottom) which are Pol II degradation-upregulated gene and active gene of RNA-seq after α-amanitin treatment for 24 h and UV irradiation with 12 h recovery, respectively. (**G**) Gene functional analysis of up-regulated genes after α-amanitin treatment for 24 h (left) and UV irradiation with 12 h recovery (right). (**H**) RPB1 pSer2 (left) and RPB1 pSer5 (right) ChIP-qPCR analysis of gene body regions and TSSs of selected Pol II degradation-upregulated genes after treatment with UV irradiation with 3 h recovery, respectively. The values were normalized to input and plotted as the means ± SEMs. Statistical significance was determined by a two-tailed *t* test with at least three replicates. *P < 0.05, **P < 0.01, ***P < 0.001. (**I**) A model illustrating our findings. Created with BioRender.com.

Given RPB1’s susceptibility to degradation under diverse external environmental stimuli (64), we proceeded to assess gene expression following treatment with α-amanitin or UV using spike-in RNA-seq. Western blotting analyses confirmed the anticipated RPB1 degradation (Figure 7A). Global gene repression was observed, alongside a subset of genes displaying increased expression (Figures 7B, Supplementary Figure S6G). Many Pol II degradation upregulated genes also exhibited increased expression after α-amanitin or UV treatment (Figures 7C, D). However, specific genes were activated uniquely by each treatment (Figures 7E, F), possibly stemming from experimental variations or other unidentified activation mechanisms. Regarding the cellular functions of genes overlapping with RPB1 deletion, UV damage, and α-amanitin exposure, such as *Popdc2*, *Apon*, and *Rnf223*, insights into their roles may shed light on cellular responses to RPB1 depletion and environmental stresses. For instance, Popdc's documented role in heart and muscle function, highlighted in studies like that of Schindler *et al.*, underscores its importance in cardiac rhythm and stress response mechanisms within these tissues (65). The upregulation of *Popdc2* after RPB1 degradation may represent an adaptive response to maintain structural integrity under transcriptional stress. *Apon* (Apoptin) is predicted to be involved in cholesterol metabolism, while the upregulation of lipid metabolism and inflammatory response genes under stress conditions is documented (66). These processes are crucial for maintaining cellular homeostasis and responding to cellular damage, which could be exacerbated by the loss of RPB1 function. *Rnf223* (Ring finger protein 223), a ubiquitin ligase, may participate in protein degradation and cellular signaling pathways (67). The upregulation of *Rnf223* post-RPB1 degradation indicates a potential need for enhanced degradation of damaged or misfolded proteins, aligning with the critical role of ubiquitination in DNA damage responses discussed by Huen *et al.*. Collectively, the upregulation of these genes suggests a complex cellular effort to adapt and respond to the compromised transcriptional landscape and resulting cellular stress.

Gene functional enrichment analyses highlighted enrichment of GPCR ligand binding and specific functions related to development or environmental stimulus in the upregulated genes following α-amanitin and UV treatments (Figure 7G). The upregulated genes following α-amanitin and UV treatments also exhibited increased pSer2 but decreased pSer5 (Figure 7H). This suggests that the mechanisms governing RPB1 degradation upregulated subsets of gene transcription elongation may also be triggered by other environmental stimuli leading to RPB1 degradation in mammalian cells.

## Discussion

We provided the first comprehensive analysis of RPB1 degradation upregulated genes indicates enrichment in GPCR ligand binding, association with polycomb, and a prevalence of less paused short genes. Furthermore, pSer2 at these upregulated genes demonstrates an increase of transcription elongation and RNA stability after RPB1 degradation but susceptibility to RPB2 degradation. We propose that this working model may also be applicable to other instances of RPB1 degradation under various environmental stresses in mammalian cells (Figure 7I). The detailed mechanisms underlying activated transcription and RNA stability in specific gene subsets following Pol II degradation warrant further investigation in the future.

The RPB1 protein, with a substantial molecular weight of 217 kDa, may exhibit different preferences for degradation at the N-terminus and C-terminus due to structural limitations or varying accessibility of degron sequences. Previous research has indicated that attaching the degron tag to the C-terminal domain of RPB1 results in only partial degradation of the C-terminal domain (CTD) (23). In such instances, the CTD-degraded Pol II retains its elongation capacity but struggles to terminate, leading to transcriptional read-through. This phenomenon could erroneously elevate the expression levels of downstream lowly expressed genes. Consequently, the presence of read-through transcripts near these genes may give the impression of upregulation. This clarifies why the CTD degron of RPB1 triggers more upregulated genes compared to the NTD degron. When the degron tag is affixed to the N-terminal domain (NTD) of RPB1, complete RPB1 degradation occurs. Both the CTD and NTD degrons of RPB1 have been shown to induce transcriptional repression in prior studies (35). To identify the genes upregulated by Pol II degradation, we cross-referenced the upregulated genes present in both the NTD and CTD degrons of RPB1, excluding those induced by transcriptional read-through from our final lists of Pol II degradation-induced upregulated genes. Following the outcomes displayed in Figure 2A, we solely utilized the NTD degron of RPB1 for the remainder of our investigation.

We observed the activation of polycomb-bound, less paused short genes following RPB1 degradation, and the detailed molecular mechanisms require further investigations. The degradation of RPB1 by auxin leads to a dramatic reduction in its protein level, though it does not eliminate it entirely; a small amount of RPB1 remains, as previously observed (29,30). It seems that RPB1 degradation is more likely to affect active genes, particularly long active genes and those may be involved in transcription-coupled DNA repair (24,61). Consequently, Pol II may not be efficiently recycled for these active long genes, while shorter, less active genes have greater opportunity for total Pol II recycling, despite a decrease in overall levels. Upon entering the elongation phase, Pol II becomes pSer2. Notably, the resistance of the pSer2 state in our upregulated genes to RPB1 depletion, but not to RPB2 depletion in mESCs. RPB1-degron in the elongating Pol II might not be as readily accessible to auxin compared to the initiating/paused Pol II. Conversely, RPB2-degron appears to be more amenable to auxin, and its depletion could structurally impact elongating Pol II, potentially rendering RPB1-degron less susceptible to degradation under such circumstances.

We observed that the increased PRO-seq signals but a notable decrease in Pol II ChIP-seq at the transcription start site (TSS) for the up-regulated genes post RPB1 depletion. These inconsistencies can be attributed to the distinct nature of PRO-seq and ChIP-seq assays, each capturing different aspects of Pol II activity and chromatin occupancy. PRO-seq data reflects the activity of actively transcribing RNA Pol II *in vitro*, capturing nascent RNA transcripts. Hence, the heightened signal at the TSSs in PRO-seq likely signifies the presence of actively transcribing Pol II complexes at the gene's TSSs. Conversely, Pol II ChIP-seq data offers a snapshot of total Pol II occupancy on chromatin *in vivo*, encompassing both phosphorylated and non-phosphorylated forms of Pol II. The observed decline in Pol II signal at the TSSs in ChIP-seq could stem from various factors, such as Pol II degradation or alterations in Pol II occupancy dynamics,

Previous studies showed that Ser5 phosphorylated Pol II CTD is associated with Polycomb complex recruitment (68–70). Studies in Schizosaccharomyces pombe and the ciliate Oxytricha trifallax have shed light on the roles of RPB2 subunit paralogs in coordinating chromatin modifications and transcriptional regulation (62,71). Although we have noted correlations between RPB1 and RPB2 depletion and specific epigenetic changes like alterations in H3K27me3, H3K4me3 and H3K9me3 levels, the causal relationships remain incompletely understood. These changes could stem directly from RPB1 or RPB2 depletion, acting through interactions with chromatin-modifying enzymes or transcription factors, or indirectly as secondary effects due to altered transcriptional activity. The dynamic and interconnected nature of epigenetic regulation underscores the need for further mechanistic studies to unravel the direct and indirect impacts of Pol II subunit depletion on chromatin modifications and transcriptional regulation. Further investigation into the crosstalk between RNA polymerase subunits and various epigenetic regulators could unveil specific connections between the RNA polymerase code and the histone or epigenetic code.

## Supplementary Material

gkae478_Supplemental_Files

## Data Availability

The raw sequence data reported in this paper have been deposited in the Genome Sequence Archive (Genomics, Proteomics & Bioinformatics 2021) in National Genomics Data Center (Nucleic Acids Res 2022), China National Center for Bioinformation / Beijing Institute of Genomics, Chinese Academy of Sciences (GSA: CRA014134) that are publicly accessible at https://ngdc.cncb.ac.cn/gsa (72,73). The mass spectrometry proteomics data have been deposited to the ProteomeXchange Consortium (http://proteomecentral.proteomexchange.org) via the iProX partner repository (74,75) with the dataset identifier PXD048023. The iProX project ID is IPX0007789000.
