# Supplementary material for: Increased transcriptional elongation and RNA stability of GPCR ligand binding genes unveiled via RNA polymerase II degradation
Source: Nucleic Acids Res. 2024 Jun 6;52(14):8165–83. doi: 10.1093/nar/gkae478 (PMC11317166; doi:10.1093/nar/gkae478)
Supplement: gkae478_Supplemental_Files [file gkae478_supplemental_files.zip › Supplementary Figures_corrected.pdf]

Figure S1

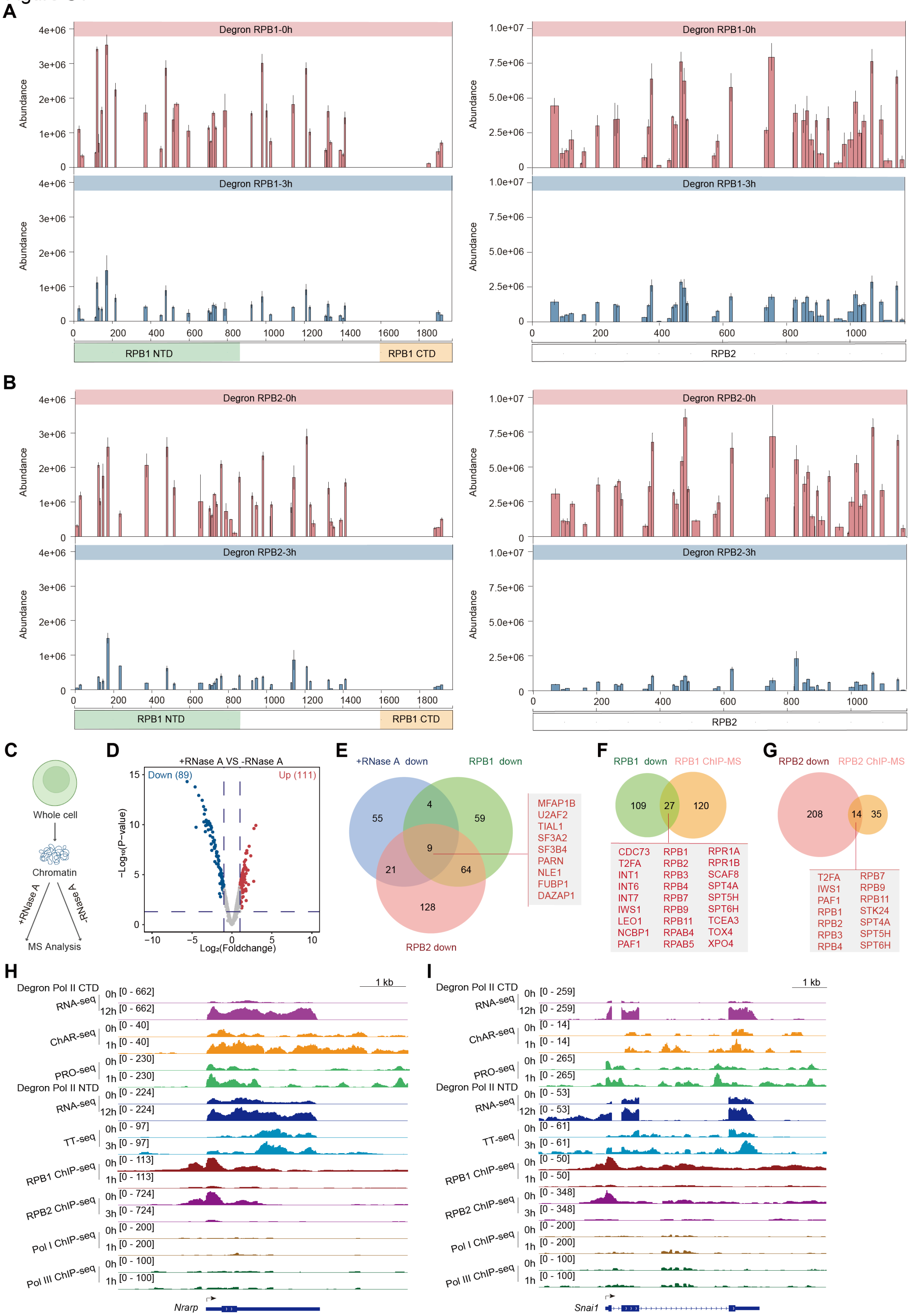
A
Degron RPB1-0h
Degron RPB1-3h
Abundance
RPB1 NTD
RPB1 CTD
RPB2
B
Degron RPB2-0h
Degron RPB2-3h
Abundance
RPB1 NTD
RPB1 CTD
RPB2
C
Whole cell
Chromatin
+RNase A
-RNase A
MS Analysis
D
+RNase A VS -RNase A
Down (89)
Up (111)
−Log₁₀(P−value)
Log₂(Foldchange)
E
+RNase A down
RPB1 down
RPB2 down
55
4
59
9
21
64
128
MFAP1B
U2AF2
TIAL1
SF3A2
SF3B4
PARN
NLE1
FUBP1
DAZAP1
F
RPB1 down
RPB1 ChIP-MS
109
27
120
CDC73
T2FA
INT1
INT6
INT7
IWS1
LEO1
NCBP1
PAF1
RPB1
RPB2
RPB3
RPB4
RPB7
RPB9
RPB11
RPAB4
RPAB5
RPR1A
RPR1B
SCAF8
SPT4A
SPT5H
SPT6H
TCEA3
TOX4
XPO4
G
RPB2 down
RPB2 ChIP-MS
208
14
35
T2FA
IWS1
PAF1
RPB1
RPB2
RPB3
RPB4
RPB7
RPB9
RPB11
STK24
SPT4A
SPT5H
SPT6H
H
Degron Pol II CTD
RNA-seq
ChAR-seq
PRO-seq
Degron Pol II NTD
RNA-seq
TT-seq
RPB1 ChIP-seq
RPB2 ChIP-seq
Pol I ChIP-seq
Pol III ChIP-seq
1 kb
Nrarp
I
Degron Pol II CTD
RNA-seq
ChAR-seq
PRO-seq
Degron Pol II NTD
RNA-seq
TT-seq
RPB1 ChIP-seq
RPB2 ChIP-seq
Pol I ChIP-seq
Pol III ChIP-seq
1 kb
Snai1

Figure S1 | RPB1 and RPB2 degradation induced degradation of Pol II on chromatin.

- **A.** Bar plot indicating peptide abundance of RPB1 and RPB2 on chromatin after RPB1 (left) and RPB2 (right) degradation, respectively. The values were plotted as the means ± SDs.
- **B.** Similar as **A**, while showing the peptide abundance of RPB1 and RPB2 on chromatin after RPB2 (left) and RPB1 (right) degradation, respectively.
- **C.** Schematic workflow to identify differential proteins on chromatin with or without RNase A treatment. Created with BioRender.com.
- **D.** Volcano plot showing protein abundance changes on chromatin after treatment of RNase A, compared with untreated samples. Differential proteins were determined with p-value < 0.05 and Log_2(Foldchange) > 1 or Log_2(Foldchange) < -1.
- **E.** Venn plot showing the overlap of down-regulated chromatin proteins identified after RNase A treated, RPB1 degradation and RPB2 degradation.
- **F.** Venn plot showing the overlap of decreased proteins identified in chromatin-MS after degradation of RPB1 and the enriched proteins identified in RPB1 ChIP-MS.
- **G.** Venn plot showing the overlap of decreased proteins identified in chromatin-MS after degradation of RPB2, and the enriched proteins identified in RPB2 ChIP-MS.
- **H.** Tracks for *Nrarp* which is a Pol II degradation-upregulated gene of different sequencing data after Pol II CTD or NTD degradation for the indicated durations.
- **I.** Tracks for *Snai1* which is a Pol II degradation-upregulated gene of different sequencing data after Pol II CTD or NTD degradation for the indicated durations.

Figure S2

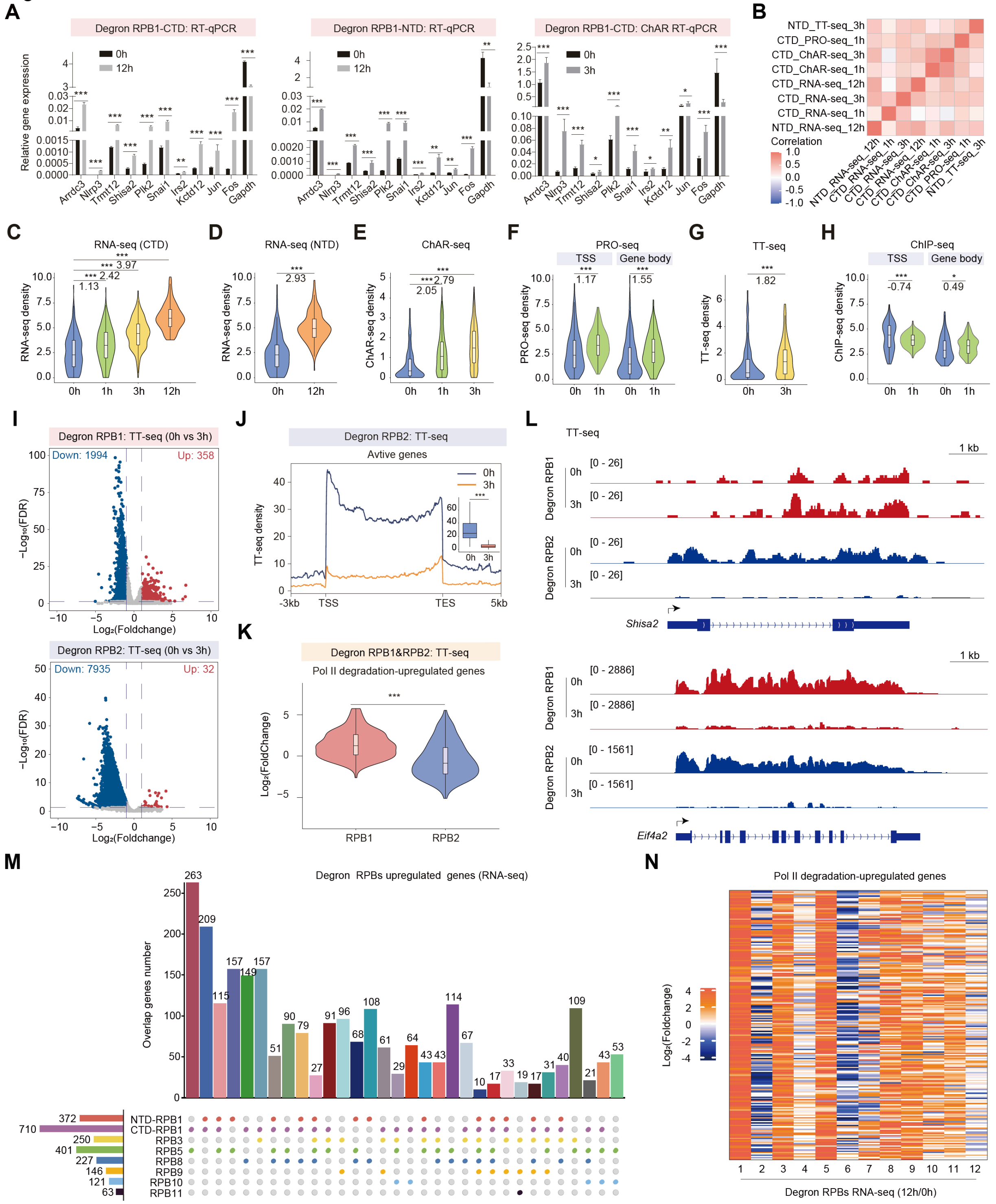

Figure S2 | RPB1 degradation induces increased expression in subsets of genes, while RPB2 degradation induces global repression.

A. RT-qPCR of selected Pol II degradation-upregulated genes from total RNA (left and middle) and chromatin associated RNA (ChAR) (right) after RPB1 CTD (left and right) and NTD (middle) degradation. The values were normalized to β-actin gene for RT-

qPCR or Drosophila_CG10433 transcript expression and plotted as the means ± SEMs. Statistical significance was determined by a two-tailed t test with at least 3 replicates. *$p < 0.05$, **$p < 0.01$, ***$p < 0.001$.

B. Heatmap indicating correlation of Pol II degradation-upregulated genes in different Pol II degradation sequencing data. The value indicating Pearson correlation coefficient was calculated.

C-H. Violin plot showing the Log_2 transformed density of RNA-seq, ChAR-seq, PRO-seq in TSSs (left) and gene body regions (right), TT-seq, Pol II ChIP-seq in TSSs (left) and gene body regions (right) of Pol II degradation-upregulated genes before (0h) and after indicated time point of RPB1 degradation, respectively. The number below significance indicated the median Log_2(Foldchange) between indicated conditions. Statistical significance was assessed by a two-sided Wilcoxon test. *$p < 0.05$, ***$p < 0.001$.

I. Volcano plots indicating TT-seq gene expression changes after RPB1 (top) and RPB2 (bottom) depletion of active genes, compared with the corresponding untreated cells. Differential expression genes were determined with adjusted p-value < 0.05 and Log_2(Foldchange) > 1 or Log_2(Foldchange) < -1.

J. Metagene profiles of normalized TT-seq reads after RPB2 degradation in active genes. The box plots showed the comparison of changes in the gene body regions. Statistical significance was assessed by a two-sided Wilcoxon test. ***$p < 0.001$.

K. Violin plot showing Log_2(Foldchange) of Pol II degradation-upregulated genes after RPB1 and RPB2 degradation. Statistical significance was assessed by a two-sided Wilcoxon test. ***$p < 0.001$.

L. Tracks for *Shisa2* (top) and *Eif4a2* (bottom) which are Pol II degradation-upregulated gene and active gene in TT-seq after degradation of RPB1 and RPB2, respectively.

M. Upset plot showing the overlap up-regulated genes after Pol II subunits degradation for 12h in RNA-seq. The bar plot at the bottom left indicated the number of up-regulated genes after corresponding Pol II subunits degradation with adjusted p-value < 0.05 and Log_2(Foldchange) > 1 for genes with RPKM > 1 in RNA-seq.

N. Heatmap showing Log_2(Foldchange) of Pol II degradation-upregulated genes through poly(A) RNA-seq analysis after individual RNA Pol II subunit depletion for 12h.

Figure S3

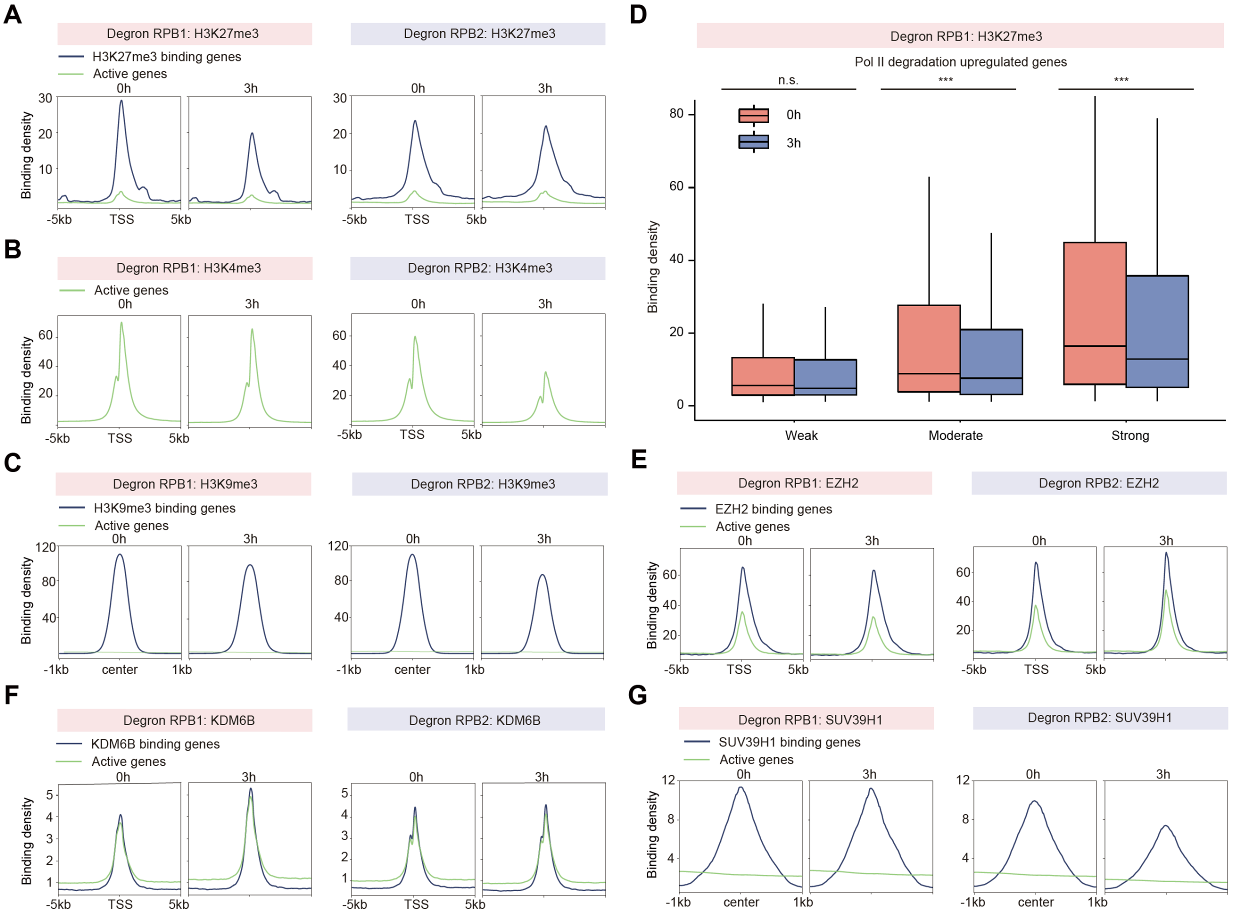

Figure S3 | Degradation of RPB1 led to a reduction in H3K27me3 density, whereas degradation of RPB2 resulted in decreased H3K9me3 density.

A. Metagene profiles of normalized binding density of H3K27me3 at the TSSs of H3K27me3 binding genes and active genes after RPB1 (left) and RPB2 (right) degradation, respectively.

B. Metagene profiles of normalized binding density of H3K4me3 at the TSSs of active genes after RPB1 (left) and RPB2 (right) degradation, respectively.

C. Metagene profiles of normalized binding density of H3K9me3 at the center of their corresponding binding genes and active genes after RPB1 (Left) and RPB2 (Right) degradation.

D. Box plot showing the H3K27me3 binding density before (0h) and after (3h) RPB1 depletion with RPB1 degradation-induced upregulated genes in TT-seq. The upregulated genes were classified in three groups according to their Log_2(Foldchange): Weak (0-1), Moderate (1-2) and Strong (>2). Statistical significance was assessed by a two-sided Wilcoxon test. n.s. not significant, ***p < 0.001.

E-G. Metagene profiles of normalized binding density of EZH2, KDM6B and SUV39H1 of their corresponding binding genes and active genes after RPB1 (left) and RPB2 (right) degradation, respectively.

Figure S4

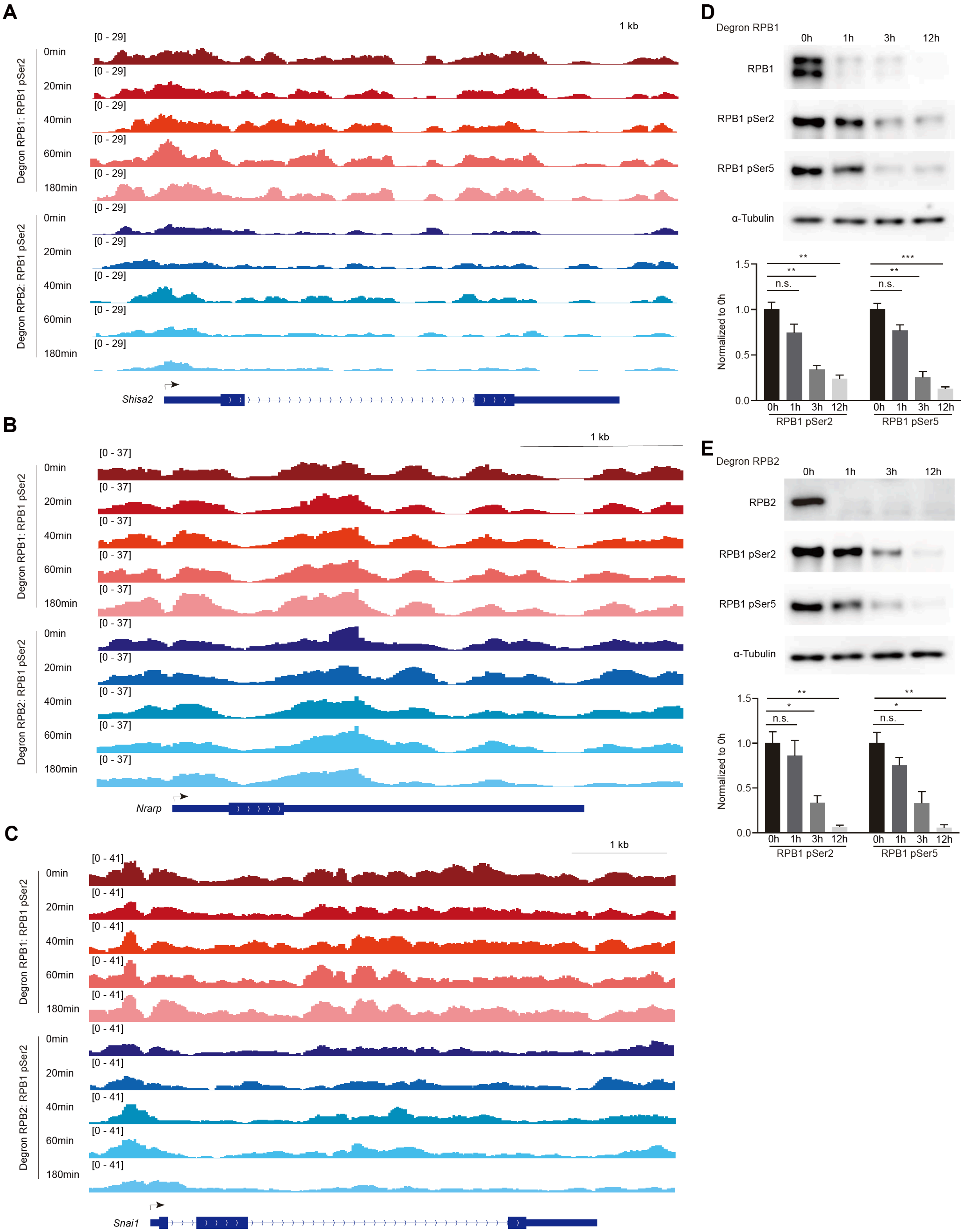

Figure S4 | The pSer2 ChIP-seq results indicated an increase after RPB1 deletion, while pSer2 continued to decrease after RPB2 depletion for the Pol II degradation upregulated genes.

A-C. Tracks (left) for *Shisa2, Nrarp, Snai1* which are Pol II degradation-upregulated gene of time-course pSer2 ChIP-seq after RPB1 (top) and RPB2 (bottom) degradation. Note: The pSer2 occupancy at *Nrarp* and *Snai1* decreased after degron RPB2 treatment for 180 minutes, despite some experimental variations observed at other time points.

D-E. Western blot analysis and quantifications of RPB1 pSer2, RPB1 pSer5 level after RPB1 and RPB2 degradation for indicated time, respectively (top). The mean intensities were normalized to 0h (bottom). Statistical significance was determined by a two-tailed t test with at least 3 replicates. n.s. not significant. *p < 0.05, **p < 0.01, ***p < 0.001.

Figure S5

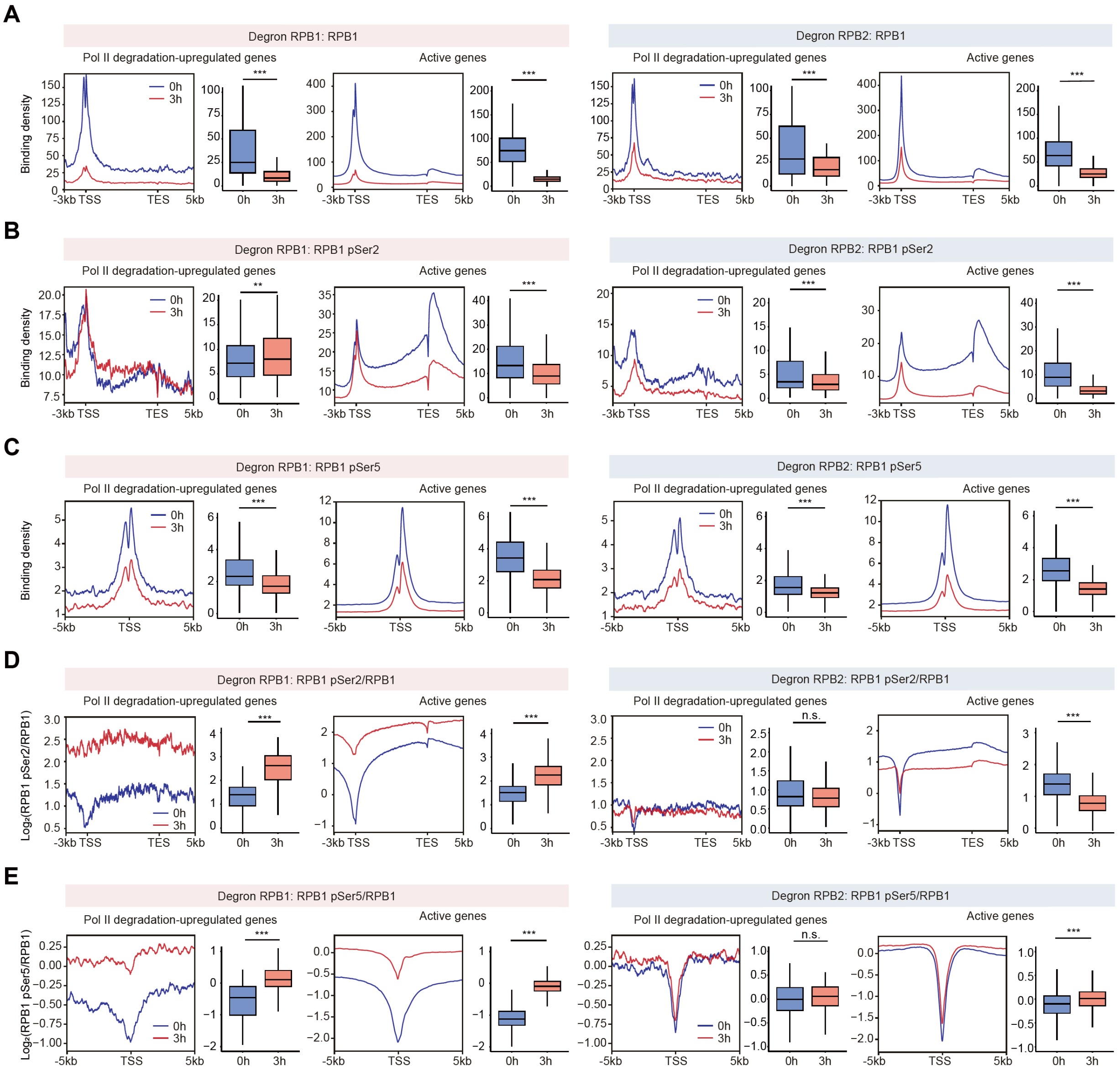

Figure S5 | An elevation in Ser2P/Pol II and Ser5P/Pol II levels for upregulated and active genes post-RPB1 depletion.

A-C. Metagene profiles of ChIP-seq of Pol II, RPB1 pSer2 and pSer5 of Pol II degradation-upregulated genes and active genes after RPB1 (left) and RPB2 (right) degradation, respectively. The box plots showed the comparison of changes of Pol II and RPB1 pSer5 in the TSS and RPB1 pSer2 in the gene body.

D-E. Metagene profiles showing the ratio of Pol II pSer2 or pSer5 ChIP-seq signal over Pol II ChIP-seq signal of Pol II degradation-upregulated genes and active genes after RPB1 (left) and RPB2 (right) degradation, respectively. Statistical significance was assessed by a two-sided Wilcoxon test. n.s. not significant. **$p < 0.01$, ***$p < 0.001$.

Figure S6

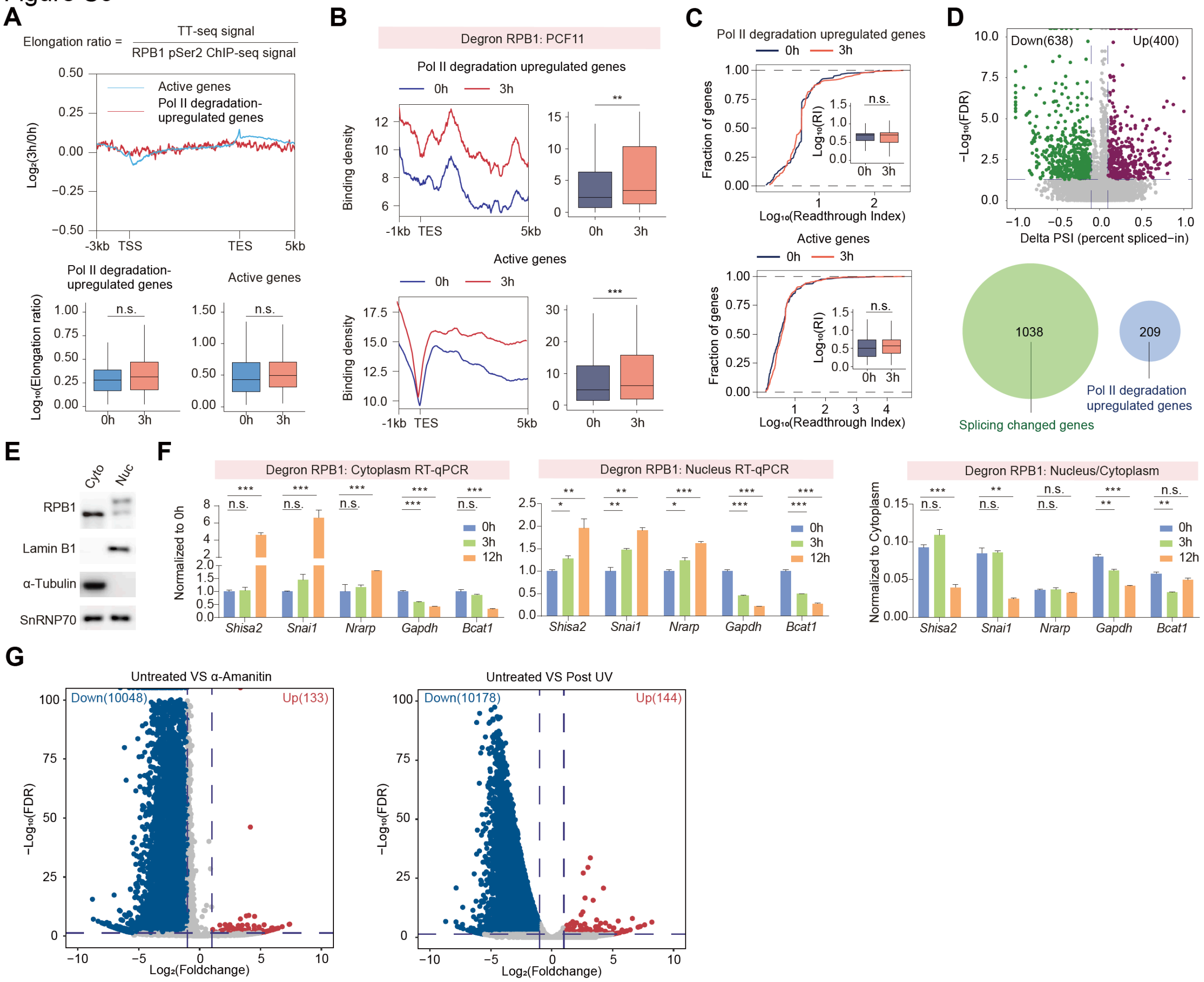

Figure S6 | Transcription termination, mRNA splicing, 3' end processing, and RNA export do not directly account for the immediate upregulation of genes following Pol II degradation.

A. Metagene profile showing the Log_2(Foldchange) of elongation ratio (TT-seq signal/Pol II pSer2 ChIP-seq) in active genes and Pol II degradation-upregulated genes after RPB1 degradation (top). The box plots showed the comparison of elongation ratio before (0h) and after (3h) RPB1 degradation in Pol II degradation-upregulated genes and active genes, respectively (down). Statistical significance was assessed using a two-sided Wilcoxon test. n.s. not significant.

B. Metagene profiles of PCF11 CUT&Tag at the TESs of Pol II degradation-upregulated genes (top) and active genes (bottom) after RPB1 degradation. The box plots showed the comparison of changes in TESs. Statistical significance was assessed by a two-sided Wilcoxon test. **$p < 0.01$, ***$p < 0.001$.

C. Empirical cumulative density function (ECDF) and violin plots showing the changes in the readthrough index of Pol II degradation-upregulated genes (top) and active genes (bottom) based on TT-seq signals. Statistical significance was assessed using a two-sided Wilcoxon test. n.s. not significant.

D. Volcano plots indicating differential exon inclusion or exon skipping events identified in RNA-seq after RPB1 degradation for 3h compared with untreated cells (top).

Differential events were determined with adjusted p-value < 0.05 and PSI > 0.1 or PSI < -0.1. Venn plot showing the overlap between splicing changed genes identified in a and Pol II degradation up-regulated genes (bottom).

E. Western blot analysis confirmed the cytoplasmic (Cyto) and nuclear (Nuc) isolations.

F. RT-qPCR of selected Pol II degradation-upregulated genes (*Shisa2*, *Nrarp*, *Snai1*), active genes (*Gapdh*, *Bcat1*) from cytoplasmic RNA (left) and nuclear RNA (right). The values were firstly normalized to Drosophila_CG10433 transcript and then normalized to 0h. The bar plots were shown as tmeans ±SEMs. Statistical significance was determined by a two-tailed t test with at least 3 replicates. n.s. not significant. *p < 0.05, **p < 0.01, ***p < 0.001. The ratio of nuclear RNA over cytoplasmic RNA (nuclear RNA/cytoplasmic RNA).

G. Volcano plots indicating RNA-seq gene expression changes after α-amanitin treatment for 24h and UV irradiation with 12h recovery, compared with the corresponding untreated cells. Differential expression genes were determined with adjusted p-value < 0.05 and Log_2(Foldchange) > 1 or Log_2(Foldchange) < -1.
